# Supplementary material for: Functional Classification and Interaction Selectivity Landscape of the Human SH3 Domain Superfamily
Source: Cells. 2024 Jan 20;13(2):195. doi: 10.3390/cells13020195 (PMC10814857; doi:10.3390/cells13020195)
Supplement: Supplementary file 1 [file cells-13-00195-s001.zip › cells-2796338-supplementary.pdf]

Supplementary information

Functional classification and interaction selectivity landscape of the human SH3 domain superfamily*

Neda S. Kazemein Jasemi, Mehrnaz Mehrabipour, Eva Magdalena Estirado, Luc Brunsveld, Radovan Dvorsky, Mohammad R. Ahmadian

Institute of Biochemistry and Molecular Biology II, Medical Faculty and University Hospital Düsseldorf, Heinrich Heine University Düsseldorf, Universitätsstrasse 1, 40225 Düsseldorf, Germany

Table S1. Proteins used in this study

SH3 domains[1]	Construct (aa)	UniProt ID
ABI1	446-505	Q8IZP0
ABL2	107-167	P42684
ABL2^{Set-1} (S121K, N124R, G155F, W156Y)		
ARHGAP12	12-74	Q8IWW6
ARHGAP12^{Set-1} (K28S, R30N, F62G, Y63W)		
ARHGAP12^{Set-2} (K31T, T46Q, A66S, Q67N)		
ARHGEF30 (OBSCN)	5600-5667	Q5VST9
BIN1	520-593	O00499
BIN1^{Set-1} (D536N, T537D, Q558D, D559P)		
CRK-1	132-192	P46108
DLG2	536-606	Q15700
GRB2-1	1-58	P62993
GRB2-2	158-215	
ITSN1-1	740-806	Q15811
ITSN1-2	913-971	
ITSN1-3	1002-1060	
ITSN1-4	1070-1138	
ITSN1-5	1155-1214	
NCK1-1	2-61	P16333
NCK1-2	106-165	
NCK1-3	190–252	
NCK1-3^{Set-1} (N205D, D206T, D226Q, P227D)	190–252	
NCK1-3^{Set-2} (N225E, W229G, K244E)	190–252	
RASA1	279–341	P20936
RIMBP3B-1	832-899	A6NNM3
SRC	77-140	P12931
SH3GLB1	305-365	Q9Y371
SH3PXD2A-1	166-225	Q5TCZ1
SORBS1-1	793-852	Q9BX66
SNX9	1-62	Q9Y5X1

[1] Expressed in the *Escherichia coli* strains CodonPlus, Rosetta, and BL21(DE3) with the use of pGEX4T-1.

Table S2. List of peptides used in this study.

Peptide name	Peptide sequence
P1[1]	1078SAPNSPRTPLTPPPAS1093
P2	1124VTLPHGPRSA1133
P3	1146EVPVPPPVPPRRRPESAPAESSPSKI1171
P4	1176LDSPPAIPPRQPTSK1190
P5	1204ISDPPESPPLLPPREPVRTPDV1225
P6	1227SSSPLHLQPPPLGKK1241
P7	1247AFFPNSPSPFTPPPPQTPSPHGT1269
P8	1271RHLPSPPLTQ1280
P9	1287IAGPPVPPRQS1297
P10	1300QHIPKLPPKTY1310
RP1[2]	1147VPVPPPVPPRRR1158
RP2[3]	13RCEAPPVPPRRERG26

[1] P represents peptides derived from SOS1.
[2] RP1 is the reference peptide1 derived from peptide 3 (P3).
[3] RP1 is the reference peptide 2 derived from WRCH1/RHOU.

Table S3. Published structures of the SH3-PRM complexes.

Fam. no.	SH3/PRM structures[1]	PRM sequence[2]	Proposed Consensus PRM (current study)	Consensus published PRM	PDB code	Ref.[3]
1	PACSIN3/TRPV4	TKGPAPNPPPILKVW	KXX(L/A)PXXP	KXXAPXXXPX	6F55	[1]
	SNX9/EEEV nsP3 peptide	AERLIPRRPAPPVPVPA RIPSPR	RX(L/A)PXXP	RXAPXXP	7OJ9	[2]
	P85A/peptide	KRPLPPLPS	RX(L/A)PXXP	LPX(L/A)P	3I5R	[3]
2	SPTAN1/P41 peptide	APSYSPPPPP	PPXPPXP	------	2JMA	[4]
	SPTAN1/P41 peptide	PPPVPP	PPXPPXP	PXPXP	3THK	[5]
3	NCK2-1/CD3epsilon	KERPPPVPNPDY	PXXDY	PXXDY	2JXB	[6]
	EPS8L1/CD3epsilon	PPVPNPDYEPIR	PXXDY	PXXDY	2ROL	[7]
	TUBA-6 (ARHGEF36-6)/NWASP	PPPALPSSAPSG	PPPXLP	PPPXLPS	4CC2	[8]
	TUBA-6 (ARHGEF36-6)/NWASP	PPPALPSSAPSG	PPPXLP	PPPXLPS	4CC7	[8]
	TUBA-6 (ARHGEF36-6)/MENA	PPPPLPSGPAYA	PPPXLP	PPPXLPS	4CC3	[8]
4	ABL1 mutant (N114A)/P17	APTYSPPLPP	PXXXPPXPP	------	4J9E	TBP
	ABL1 mutant (H59Q-N96T)/P17	APTYSPPLPP	PXXXPPXPP	------	4J9C	TBP
	ABL1/P17	APTYSPPLPP	PXXXPPXPP	------	4J9I	TBP
	ABL1/P7	APTYPPPPPP	PXXXPPXPP	------	4J9G 4J9H	TBP
	ABL1 mutant (N114A)/P41	APSYSPPPPP	PXXXPPXPP	PXXP	2O88	[9]
	ABL1/P41 peptide	APSYSPPPPP	PXXXPPXPP	PXXP	1BBZ 3EG1	[10, 11]
	ABL1 mutant (N114A)/P0	APTYPPPLPP	PXXXPPXPP	------	4J9D	TBP
	ABL1/P0	APTYPPPLPP	PXXXPPXPP	------	4J9F	TBP
	ABL1/3BP-1	APTMPPPLPP	PXXXPPXPP	PXXXPPXPP	1ABO	[12]
5	NCF1-2(p47phox)/p22phox	QPPSNPPPRPP	PXPXXP	PXPXXP	1OV3	[13]
	NCF1-2(p47phox)/p22phox	GPLGSKQPPSNPPPRP PAEARKKPS	PXPXXP	PPPRPPAEAR	1WLP	[14]
7	CRKII-1 (CRK-1)/C3G	DNSPPPALPPKKRQSY	PXXPX(K/R)	------	5L23	TBP
	CRK-1 (C-CRK)/C3G	PPPALPPKKR	PXXPX(K/R)	PXLPXK	1CKA	[15]
	CRKII-1 (CRK-1)/C-ABL	YEKPALPRKR	PXXPX(K/R)	PXLPXK	5IH2	[16]
	CRK-1/peptide inhibitor	YEVPGPVPPRRR	PXXPX(K/R)	PXXPXR	1B07	[17]
	CRK-1 (C-CRK)/SOS peptide	PPPVPPRR	PXXPX(K/R)	PXXPXR	1CKB	[15]
	HCK/synthetic peptide	HSKYPLPPLPSL	(K/R)XPXXP	LPX(L/A)P	2OJ2 2OI3	[18]
	FYN/synthetic peptide	VSLARRPLPPLP	(K/R)XPXXP	RXPXXP	4EIK	[19]
	FYN/synthetic peptide	APPLPPRNRPRL	PXXPXX(K/R)	PXXPXXR	4ZNX	[19]
	FYN/3BP-2	PPAYPPPPVP	PXXP	------	1FYN	[12]
	FYN/P2Lsynthetic peptide(PI3K-P85)	PPRPLPVAPGSSKT	(K/R)XXPXXP	RPLPVAP	1A0N 1AZG	[20]
	FYN/NS5A	APPIPPPR	PXXPX(K/R)	XPXXPX(K/R)	3UA7	[21]
	LYN/TIP	WDPGMPTPPLPPRPAN LGERQA	PXXPX(K/R)	PPLPPR	1WA7	[22]
	SRC/VSL12	VSLARRPLPPLP	(K/R)XXPXXP	RXLPPXP	1QWF	[23]
	SRC(C-SRC)/APP12	APPLPPRNRPRL	PXXPX(K/R)	XPPLPXR	1QWE	[23]
	SRC mutant (T98D)(C-SRC)/APP12	APPLPPRNRP	PXXPX(K/R)	XPXXPXR	4HVU	[24]
	SRC mutant (T98E)(C-SRC)/APP12	APPLPPRNRP	PXXPX(K/R)	XPXXPXR	4HVV 4HVW	[24]
	SRC/tyrosine phosphatase PEP	IPPPLPERTPESFIVVEE	PXXPX(K/R)	PXXPXR	1JEG	[25]
	SRC(C-SRC)/NL1	PLPPLP	PXXP	PXXP	1NLO	[26]
	SRC(C-SRC)/NL2	PLPPLP	PXXP	PXXP	1NLP	[26]
	SRC(C-SRC)/PLR1	AFAPPLPRR	PXXPX(K/R)	XPPLPXR	1PRM 1PRL	[27]
	SRC(C-SRC)/PLR2	RALPPLPRY	(K/R)XXPXXP	RXLPPLP	1RLP 1RLQ	[27]
	SRC(C-SRC)/NS5A	APPIPPPR	PXXPX(K/R)	PXXPXR	4QT7	[28]
8	ITSN1-2/synthetic peptide	WRDSSGYVMGPW	Exceptional	[W/F][R/W]XSX[A/G][F/Y] [L/V]XGP[W/L]	4IIM	[29]
	ITSN2-2/synthetic peptide	WRGSLSYLKGPL	Exceptional	[W/F][R/W]XSX[A/G][F/Y] [L/V]XGP[W/L]	4IIO	[29]
	betaPIX (ARHGEF7)/alphaPAK	DATPPPVIAPRPEHTKS VYTRS	PXXXPR	XPXXXPXR	1ZSG	[30]
	betaPIX (ARHGEF7)/CBL-b	RPPKPRPR	PXXXPR	PXXXPR	2AK5	[31]
	betaPIX(ARHGEF7)/AIP4	GGFKPSRPPPRPSRPPP PTPRRPASV	PXXXPR	PXXXPR	2P4R	[32]
	betaPIX (ARHGEF7)/ITCH	GSGGGKPSRPPPRPSRP PPPTPRRPASV	PXXXPR	PXXPXXR	5SXP	[33]
	betaPIX (ARHGEF7)/PAK2	PPVIAPRPEHTKSIYTRS	PXXXPR	PXXXPR	2DF6	[34]
	IRTKaS(BAIAP2L1)/EspFu-R47	HIPPAPNWPAPTPPVQ N	PXXXP	IPxZPxxxZPxZP (wherein Z is P, A, I, L, or V)	2KXC	[35]

	PLCG1/SLP-76	QPPVPPQRPM	PXXXPXR	XPXXPXRP	1YWO	[36]
9	GRB2-1 mutant (Y7V,C32S)/SOS1	VPPPVPPRRR	PXXPX(K/R)	------	1AZE	[37]
	GRB2-1/SOS1	VPPPVPPRRR	PXXPX(K/R)	------	1GBQ 3GBQ 4GBQ	[38]
	DOCK2/ELMO1	RLLDLENIQIPDAPPPIP KEPSNYDFVY	PXXPX(L/P)	------	2RQR	[39]
	DOCK2/ELMO1	PDAPPPIP	PXXPX(L/P)	------	3A98	[39]
	p67^{phox}-2 (NCF2-2)/p47^{phox} (NCF1)	SKPQPAVPPRPSADLIL NRCSESTKRKLASAV	PXXPX(K/R)	PXXPXR	1K4U	[40]
	P40^{phox} (NCF4)/p47^{phox} (NCF1)	KPQPAVPPRPSAD	PXXPX(K/R)	------	1W70	[41]
	Cortactin (SRC8)/AMAP1	KRPPPPPPG	PXXPX(L/P)	RXXPXXP	2D1X	[42]
	Cortactin (SRC8)/Arg nonreceptor tyrosine kinase	SSVVPYLPRLPIL	PXXPX(L/P)	------	3ULR	[43]
	Ponsin-2(SORBS1-2)/Paxillin	VPPPVPPPPS	PXXPX(L/P)	------	2O9V	[44]
	CAP-2 (SORBS1-2)/Vinculin	ELAPPKPPLPE	PXXPX(L/P)	XPXXPXL	4LN2	[45]
	CAP-1(SORBS1-1)/Vinculin	VPPPRPPPPE	PXXPX(L/P)	XPXXPXX	4LNP	[45]
	NEBL/XIRP2	PPPTLPKPKLPKH	PXXPX(L/P)	PPXXXPKP	4F14	[46]
10	GRB2-2/synthetic peptide	RHYRPLPPLP	RXX(K/R)P	------	1IO6	TBP
	GRB2-2/SOS1 peptide	APPPRPPKP	RXX(K/R)P	RXXKP	2W0Z	[47]
	GRB2-2/Gab2	IQPPPVNRNLKPDR	RXX(K/R)P	PXXXRXXKP	2VWF	[48]
	CD2AP-2/ARAP1	PTPRPVPMKRHIFR	PX(P/A)XXR	PX(P/A)XXR	4X1V	[49]
	CD2AP-2/RIN3	TAKQPPVPPPRKKRIS	PX(P/A)XXR+ PXXPX(K/R)	PX(P/A)XXR	3U23	[49]
	CD2AP-1/RIN3	AKKNLPTAPPRRRVSE	PX(P/A)XXR + PXXPX(K/R)	PX(P/A)XXR	4WCI	[49]
	CD2AP-1/CBL-B	PPKPRPRR	PX(P/A)XXR	PXXXPR	2J6F	[50]
	CMS-1(CD2AP1-1)/CD2	PLPRPRV	PX(P/A)XXR	PXXXPR	2J6O	[50]
	CMS-1(CD2AP1-1)/CD2	KGPPLPRPRV	PX(P/A)XXR	PXXXPR	2J7I	[50]
	CIN85-1(SH3KBP1-1)/CBL-b	PARPPKPRPRR	PX(P/A)XXR + RXX(K/R)P +PXXPX(K/R)	PXXXPR	2BZ8	[31]
	STAM2/AMSH	AKPPVVDRSLKPGA	RXX(K/R)P	PX(V/I)(D/N)RXXKP	5IXF	[51]
	STAM2/UBPY-derived peptide	TPMVNRENKPP	RXX(K/R)P	PX(V/I)(D/N)RXXKP	1UJ0	[52]
	BIN1/C-MYC	LLPTPPLSPSRRSG	PXXPX(K/R)	PXXPXR	1MV0	[53]
	GRAP2-2 (Mona/Gads)/HPK1	GQPPLVPPRKEKMRGK	PXXPX(K/R)	PXVPXRXXK	1UTI	[54]
	GRAP2-2 (Mona/Gads)/ phosphatase-like protein HD-PTP	PPPRPTAPKPLL	PXXPXXP(K/R)	RXXXXK	2W10	[48]
	GRAP2-2/Lymphocyte cytosolic protein2(SLP-76)	APSIDRSTKPPL	RXX(K/R)P	PXXDRXXKP	1OEB 1H3H	[55, 56]
	GRAP2-2/SLP-76	PSIDRSTKP	RXX(K/R)P	PXXXRXXKP	2D0N	[57]
	ASAP1/MICAL1	GPGSEPPPKPPRS	PXXPX(K/R)	XPXKPXR	8HLO	[58]
	STAC2/CaV1.1	EPEIPLSPRP	PXXPXXP(K/R)	------	6B27	[59]

1 Names in parentheses represent aliases.
2 The underlined residues in the second column represent motifs associated with the published consensus PRMs, while the residues highlighted in green represent the proposed consensus PRM identified in the current study.
3 TBP stands for to be published. This means that the molecular structure is available in the Protein Data Bank (PDB), but the corresponding research article is not yet publicly available.

Table S4. Published dissociation constants (K_d) determined for the SH3-PRP interactions

Fam. no.	SH3DCP	PRM	peptide Sequence[1]	proposed Consensus PRM (current study)	consensus published PRM	K_d (μM)	Method[2]	Ref.
1	SNX9	EEEV nsP3	AERLIPRRPAPPVPVPARIPSPR	RX(L/A)PXXP	RXAPXXP	0.3	ITC	[2]
	PACSIN1	Itch	PEDAGAGENRRVSGNNSPSLSNGGFKPSRPPRPSRPPPPTPRRPASVNGSPSATSESDGSSTG	RXXPXXP	K/RXXPXXPXK/R	4.33	ITC	[60]
		TRPV4	TKGPAPNPPPVLKV	KXX(L/A)PXXP	KXXAPXXXPX	51.6	HSQC	[61]
	PACSIN2	TRPV4	TKGPAPNPPPVLKV	KXX(L/A)PXXP	KXXAPXXXPX	12.7	HSQC	
	PACSIN3	TRPV4	TKGPAPNPPPVLKV	KXX(L/A)PXXP	KXXAPXXXPX	68.6	HSQC	
	p85A	Synthetic peptide	RKLPPRPSK	RX(L/A)PXXP	RXLPPRPXX	9.1	FL	[62]
		PD1R	HSKRPLPPLPSL	RX(L/A)PXXP	LPX(L/A)P	40	SPR	[3]
		PD1	HSKYPLPPLPSL	KXX(L/A)PXXP	------	120		
2	SPTAN1	Peptide41	ASYPPVPPP	PPXPPXP	------	160	FL	[63]
3	NCK1-1	N-WASP	1.LRRQAPPPPPPS<br>2.APPPPPPSRGG<br>3.GPPPPPARGRGA<br>4.TAAPPPPPPSRP<br>5.SAPSGPPPPPPSVL	PPPPP	------	>1 MM	HSQC	[64]
	EPS8	E3b1	PPPPPVDYEDEE	PPPPP+PXXDY	PXXDY	35	ELISA	[65]
	EPS8L1	CD3ε	PPVPNPDYEPIR	PXXDY	PXXDY	24	ITC	[7]
	ITK	TSAD	LLRPKPPIPAKPQLP	PXXPXLP	------	150 mM	HSQC	[66]
			LLRPKPPIPAKPQLPPEVYTIPVPRHR	PXXPXLP	------	123 mM		
4	ABL1	P4	APSYSPPPPP	PXXXPPXPP	------	1.5	FL	[67]
		P4	APTYSPPPPP	PXXXPPXPP	------	0.4		
		P8	APTYPPPAPP	PXXXPPXPP	------	5±		
		3BP-1	RAPTMPPPLPP	PXXXPPXPP		34		
5	SH3PXD2B-1/2	SH3PXD2B	GSHMGDAKQRSPKMRQRPPPRRDMTIPRGLNLPKPPIPPQVE	PXPXXP	PPPRR	15<br>11	MST<br>FL	[68]
	NCF1-2 (p47phox)	p22phox	QPPSNPPPRPPAEAR	PXPXXP	------	8.67	FL	[14]
			QPPSNPPPRPPAEARKKPSE	PXPXXP	RKKPSE	0.64		
7	CRK-1	C3G	PPPALPPKKR	PXXPX(K/R)	PXXPXK	1.9	FL	[15]
			PPPALPPKKR	PXXPX(K/R)	XXPXLPXKXX	1.89	FL	[69]
			DNSPPPALPPKKRQSAPS	PXXPX(K/R)	PXLPXK	~2	ITC	[70]
		DOCK180	DVADVPPPLPLKGSVADYGNLMENQDLLGSPTPPPPPPHQRHLPPPLPSKT	PXXPX(K/R)	PPXLPXK	0.35	SPR	[71]
		ST12	SLPGPLTPVAEGQEIGMNTETSGTSAREKELSPPPGLPSKIGSISRQSSL	PXXPX(K/R)		0.91		
		SOS1	YEVPPPVPPRRR	PXXPX(K/R)	PXXPXR	6	FL	[17]
			PPPVPPRRRR	PXXPX(K/R)		5.2	FL	[15]
	SRC	VSL12	VSLARRPLPPLP	(K/R)XPXXP	+XPpXP	0.45	FL	[23]
		APP12	APPLPPRNRPRL	PXXPX(K/R)	XPpXP+	1.2		
		Synthetic peptide	RALPPLPRY	(K/R)XXPXXP	RXLPPLPRX	7.8	FL	[62]
		SOS1	YEVPPPVPPRRR	PXXPX(K/R)	PXXPXR	25	FL	[17]
	HCK	Nef	PVRPQVPLRPMT	PXXPX(K/R)	PXXP	91	SPR	[72]
	FYN	Nef	PVRPQVPLRPMT	PXXPX(K/R)	PXXP	202	SPR	[72]
		PI3K-p85α	KRISPPTPKPRPPR	(K/R)XXPXXP	------	3 mM	HSQC	[73]
			PPRPLPVAPGSSKA	(K/R)XXPXXP	RXXPXXP	50		
			PPRPTPVAPGSSKA	(K/R)XXPXXP	RXXPXXP	300		
		P2L	PPRPLPVAPGSSKT	(K/R)XXPXXP	------	50<br>28<br>16	NMR<br>CD<br>ITC	[20]
	LCK	Tip	ATLDPGMPTPPLPPRPANLG	PXXPX(K/R)	------	16.80	FL	[74]
		TSAD	LLRPKPPIPAKPQLP	PXXPX(K/R)	------	69 mM	HSQC	[66]
			LLRPKPPIPAKPQLPPEVYTIPVPRHR	PXXPX(K/R)	XPXPXX(R/K)	161 mM		
8	βPIX (ARHGEF7)	Itch	KPSRPPRPSRPPPPTPRRPAS	PXXXPR	RPXPPXPR	1.59	ITC	[33]
			PEDAGAGENRRVSGNNSPSLSNGGFK	PXXXPR	K/RXXPXXPXK/R	1.44	ITC	[60]

			PSRPPRPSRPPPPTPRRP ASVNGSPS ATSESDGSSTG					
		PAK2	EETAPPVIAPRPDHTKSIY TRSVI	PXXXPR	PXXXPR	1.05	ITC	[34]
	ITSN1-2	Synthetic peptide	WRDSSGYVMGPW	Exceptional	[W/F][R/W]xSx[A/G][F/Y][L/V]xGP[W/L]	53	ITC	[29]
	NCK1-2	N-WASP	GPPPPPARGRGA	PXXXPXR	------	147	HSQC	[64]
			VAVPPPPPNRMY	PXXXPXR		199		
	PLCG1	SOS1	AAPVPPPVPPRRRP	PXXXPR	------	0.20mM	SPR	[75]
			AADSPPAIPPRQPT	PXXXPR		0.40mM		
			AAESPPLLPPREPV	PXXXPR		0.70mM		
			AAIAGPPVPPRQST	PXXXPR		0.28mM		
	BAIAP2L1 (IRTKS)	EspF$_U$R4$_5$	IPPAPNWPAPTPP	PXXXP	------	0.5 nM	ITC	[76]
**9**	SORBS2-1	Synthetic peptide	LRTGEAYLRYVD	Exceptional	XRXGXAYLXYVX	38	ITC	[29]
		Synthetic peptide	RLPLRPPLPHTS	PXXPX(L/P)	PXXPXXP	121	ITC	[29]
	GRB2-1	C3G	PPPALPPKKR	PXXPX(K/R)	PXXPXK	142	FL	[15]
		SOS1	PPPVPPRRRR	PXXPX(K/R)	PXXPXR	3.5	FL	
			VPPPVPPRRR	PXXPX(K/R)	PXXPPR	5.6	FL	[77]
			PVPPPVPPRRRP	PXXPX(K/R)	PPVPPR	38.64	ITC	[78]
			PVPPPVPPRRRP	PXXPX(K/R)	PX(V/L/I)PXR	39	ITC	[79]
			DSPPAIPPRQPT			55		
			ESPPLLPPREPV			117		
			IAGPPVPPRQST			82		
			YEVPPPVPPRRR	PXXPX(K/R)	PXXPXR	5	FL	[17]
			PKPLPRFPKK	PXXPX(K/R)	PXPXPRXPKK(S suggested core: PXXPXK)	250	NMR	[47]
			PVPPPVPPRRRP	PXXPX(K/R)	PXXPXR	37	NMR	
			PSPHGTRRHLPSPP	Exceptional	RR	208	NMR	
			APNSPRTPLTPPPAYS	PXXPX(L/P)	PXXPRXPXXP	280	NMR	
	SH3GL2 (Endophilin-A1)	Itch	PEDAGAGENRRVSGNNS PSLSNGGFK PSRPPRPSRPPPPTPRRP ASVNGSPS ATSESDGSSTG	PXXPX(K/R) + PXXPX(L/P)	(K/R)XXPXXPX(K/R)	0.457	ITC	[60]
**10**	STAM2	UBPY	TPMVNRENKPP	RXX(K/R)P	PX(V/I)(D/N)RXX KP	27	FL	[52]
	NCK1-3	N-WASP	VAVPPPPPNRMY	PX(P/A)XXR	------	~1mM	HSQC	[64]
			1.NRMYPPPPPALP 2.SAPSGPPPPPPSVL 3.VAPPPPPPPPPPPG 4.PGPPPPPGLPSD	PPPPP	------	>>1 mM	HSQC	
	AMPH	Dynamin-I	PSRPNR	PXXPX(K/R)	PXRPXR(H)R(H)	0.19	γ-radiation	[80]
	GRB2-2	SOS1	PVPPPVPPRRRP	PX(P/A)XXR + PXXPX(K/R)	PPVPPR	117	ITC	[78]
			PVPPPVPPRRRP	PX(P/A)XXR + PXXPX(K/R)	PX(V/L/I)PXR	125	ITC	[79]
			DSPPAIPPRQPT	PXXPX(K/R)		1,396		
			ESPPLLPPREPV	PXXPX(K/R)		1.718		
			IAGPPVPPRQST	PXXPX(K/R)		1.318		
			PVPPPVPPRRRP	PX(P/A)XXR + PXXPX(K/R)	PXXPXR	142	NMR	[47]
			PKLPPKTYKREH	PXXPX(K/R)	PXXPXKXXXKR	156		
		SLP-76	PAPSIDRSTKPPL	RXX(K/R)P	PX3RX2KP	9.7	ITC	[55]
		Gab2b	IQPPPVNRNLKPDRK	PX(P/A)XXR + RXX(K/R)P	PX3RX2KP	17.4	ITC	[48]
	GRAP2-2	SLP-76	PAPSIDRSTKPPL	RXX(K/R)P	PX3RX2KP	0.181	ITC	[55]
			APSIDRSTKPP	RXX(K/R)P	PX3RX2KP	0.675	ITC	
			PSIDRSTKP	RXX(K/R)P	PX3RX2KP	30	ITC	[57]
	BIN1	Tau	SRTPSLPTPPTREPKKVA VVRTPPKSPSSAK	PX(P/A)XXR + PXXPX(K/R)	PXPPXR and RXPPXXP	44	NMR	[81]
	STAC1	CaV1.1	EDEPEIPLSPRPRP	PXXPXXP(K/R)	------	3.92	ITC	[59]
			NVNEVKDPYPSADFPGDD EEDEPEIPLSP RPRPLAELQLKEKAVPIPE	PXXPXXP(K/R)		0.78		
	STAC2					1.85		
			EDEPEIPLSPRPRP	PXXPXXP(K/R)		9.31		
		CaV1.2	NENEDKSPYPNPETTGEE DEEEPEMPVGP	PXXPXXP(K/R)		19.3		

			RPRPLSELHLKEKAVPMPE					
	CD2AP-1 (CMS)	CD2	QKGPPLPRPRVQPKPPHG	PX(P/A)XXR	PXXXPR	100	SPR	[50, 82]
	SH3KBP1-1 (CIN85)							

[1] The underlined residues in the second column represent motifs associated with the published consensus PRMs, while the residues highlighted in green represent the proposed consensus PRM identified in the current study.
[2] CD: Circular Dichroism Spectroscopy FL: fluorescence-based titrations; HSQC: Heteronuclear Single Quantum Coherence; ITC: Isothermal titration calorimetry; MST: microscale thermophoresis

Table S5. PRM classification and occurrence in SOS1 PRD.

No.	ID	Consensus sequences	Ref.	Peptides[1]											
				P1	P2	P3	P4	P5	P6	P7	P8	P9	P10	RP1	RP2
**1**	0X1	PPPP	[83]	-	-	-	-	-	-	+	-	-	-	-	-
**2**	0X2	XPPX	[84]	+	-	+	+	+	+	+	+	+	+	+	+
**3**	1X1	PXP	[85]	+	-	+	-	-	+	+	+	+	-	+	+
**4**	1X2	PXPXP	[86]	-	-	+	-	-	-	-	-	-	-	+	-
**5**	1X3	PPXPP	[87]	-	-	+	+	+	-	-	-	+	-	+	+
**6**	2X1	PXXDY	[7]	-	-	-	-	-	-	-	-	-	-	-	-
**7**	2X2	PXXP	[62]	+	+	+	+	+	-	+	+	+	+	+	+
**8**	2X3	PXXPX[KR]	[88]	-	-	+	+	+	-	-	-	+	-	+	+
**9**	2X4	[KR]XXPXXP	[88]	-	-	-	-	-	-	-	+	-	-	-	-
**10**	2X5	PXXPXXP	[89]	+	-	+	-	-	-	-	-	-	-	+	-
**11**	3X1	PXXXP	[90]	+	-	+	+	+	+	+	-	+	+	+	+
**12**	3X2	PXXXPXXXP	[91]	-	-	+	+	+	-	-	-	-	-	-	-
**13**	3XP	PXXXPR	[92]	-	-	+	+	+	-	-	-	+	-	+	+
**14**	4XP	PXXXXP	[93]	+	-	+	+	+	-	+	-	-	-	+	-

[1] The amino acid sequences of the peptides are listed in Table S2. + Presence of consensus sequence in peptides; - Absence of consensus sequence in peptides.

Table S6. Dissociation constants (K_d)[1] for the SH3-PRP interactions determined in this study.

SH3 Domains[2,3]	Peptides[4]											
	P1	P2	P3	P4	P5	P6	P7	P8	P9	P10	RP1	RP2
ABI1	-	60.7	-	24.6	-	-	-	-	-	-	-	-
ABL2	-	-	-	125	-	-	67.3	-	-	-	11	-
ARHGAP12	-	-	13.8	-	-	-	0.2	-	-	-	16.7	15.5
ARHGEF30	-	-	-	-	-	-	8.9	-	-	-	-	-
BIN1	-	-	-	-	-	-	12.0	-	47.0	-	-	48.0
CRK-1	-	-	12.9	-	-	-	18.1	-	-	-	-	-
DLG2	-	-	-	-	-	-	58.5	-	-	-	-	-
GRB2-1	-	-	15.0	60	62	-	-	-	-	-	11.0	20.0
GRB2-2	-	-	12.0	20	35	-	-	-	-	-	3.4	12.9
ITSN1-1	-	-	-	6.6	-	-	-	-	-	-	39	11.0
**ITSN1-2**	-	-	-	-	-	-	-	-	-	-	-	-
**ITSN1-3**	-	-	-	-	-	-	-	-	-	-	-	-
**ITSN1-4**	-	-	-	-	-	-	-	-	-	-	-	-
ITSN1-5	23.0	-	-	12.0	-	-	-	-	-	-	-	23.0
NCK1-1	-	-	-	-	-	-	-	-	-	-	-	-
NCK1-2	-	-	-	-	-	-	-	-	-	-	2.0	1.0
NCK1-3	-	-	-	-	-	-	-	-	0.9	-	24.6	2.5
**NPHP1**	-	-	-	-	-	-	-	-	-	-	-	-
**RASA1**	-	-	-	-	-	-	-	-	-	-	-	-
RIMBP3B-1	-	21.0	-	-	-	-	15.0	-	-	-	-	-
**SH3GLB1**	-	-	-	-	-	-	-	-	-	-	-	-
SH3PXD2A-1	-	44.0	18.0	-	-	-	-	-	-	-	-	21.0
**SNX9**	-	-	-	-	-	-	-	-	-	-	-	-
SORBS1-1	-	-	-	-	-	-	13.2	-	-	-	-	-
SRC	-	-	2.0	-	-	-	13.3	-	-	-	-	-

[1] The dissociation constants (K_d) were determined by analyzing the fluorescence polarization data (Figure S6) shown as bar charts in Figure 2B. The evaluated K_d values were categorized into different affinity levels: high affinity (0.1 to 1.0 µM; green), intermediate affinity (1.1 to 5.0 µM; blue), low affinity (5.1 to 25 µM; red), and very low affinity (26 to 125 µM; black). No binding is indicated by a dash (-).

[2] SH3DCPs with two or more SH3 domains are indicated by a dash followed by the SH3 domain number.

[3] Proteins in bold: Seven proteins did not bind to any of the 12 peptides that were tested under the conditions of this study.

[4] Amino acid sequences of the peptides are provided in Table S2.

Table S7. Proteins containing PRMs homologous to peptides 2-9 derived from the SOS1 PRD.[1]

Abbreviation/Alias	Protein Names	Accession no.
CCDC144A	Coiled-coil domain-containing protein 144A	XP_016880918.1
DCAF1/VPRBP	DDB1- and CUL4-associated factor 1/Vpr (HIV-1) binding protein (VPRBP)	NP_001336097.1
DLGAP1/2/4	Disks large-associated protein 1/2/4	NP_001385456.1, NP_001333739.1, NP_055717.2
HMCN2	Hemicentin-2	XP_011516769.1
IQSEC2	IQ motif and SEC7 domain-containing protein 2	NP_001104595.1
MACF1	Microtubule-actin cross-linking factor 1	NP_001384402.1
MAGED4	Melanoma antigen family D, 4	EAW62887.1
NFATC2IP	NFATC2-interacting protein	NP_116204.3
PI3KAP1	Phosphoinositide-3-kinase adaptor protein 1	NP_689522.2
PLA2	Phospholipase A2	BAD92387.1
SLX4/BTBD12	Structure-specific endonuclease subunit/ BTB (POZ) domain containing 12	NP_115820.2
SSTR5	Somatostatin receptor subtype 5B	ABE27002.1
WRCH1/RHOU	Wnt-responsive CDC42 homologue/RHO-related GTP-binding protein	NP_067028.1
ZNF41	Zinc finger protein 41	NP_001311071.1
ZNF74	Zinc finger protein 74	KAI2596768.1

[1] See Figure 4 for more details.

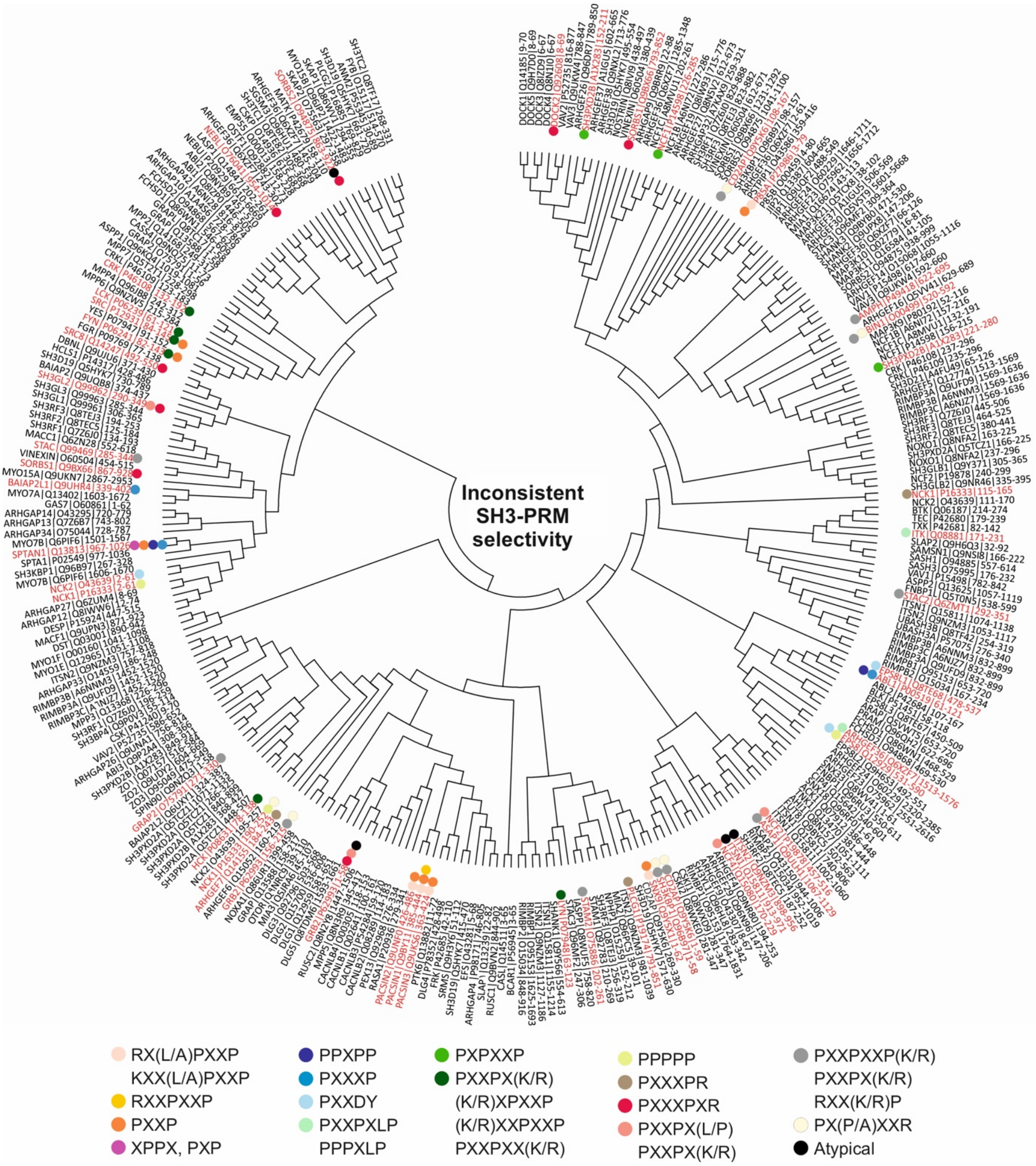

Figure S1. Evolutionary sequence-structure-function relationships of SH3 domains. A whole-sequence phylogenetic tree (tree #1) encompassing 298 human SH3 domains was constructed using the MEGA software (version 10.2.6). Using the structures and biochemical information of SH3 domains, presented in Tables S3 and S4, the interactions between PRMs and their corresponding SH3s are visually represented in the tree. The distinct preferences of SH3 domains for specific PRMs are represented by colored circles, each denoting a PRM preference, while the corresponding SH3 domains are highlighted in red. Interestingly, the PRMs exhibit clustering patterns that are inconsistent with established SH3 domain families.

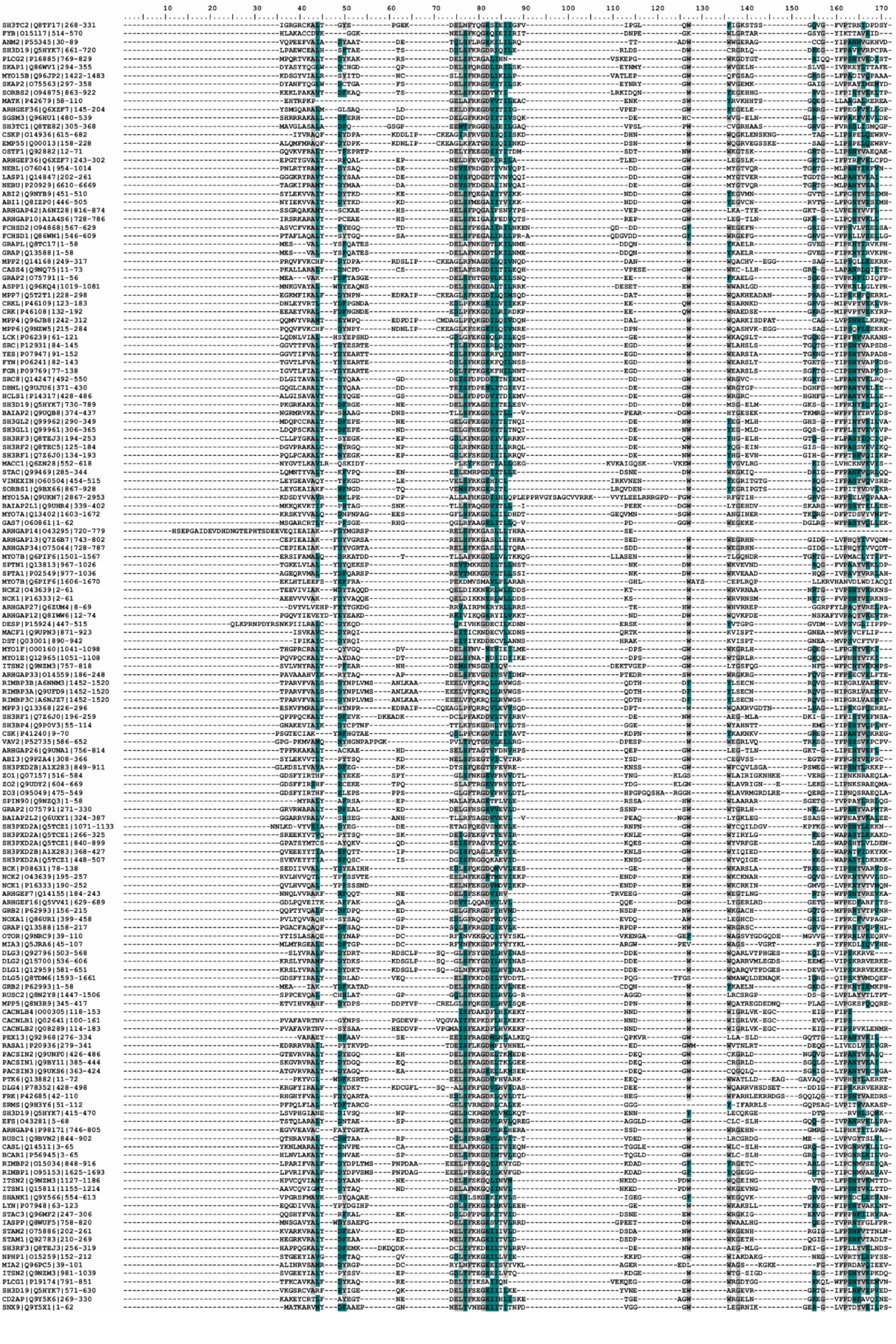

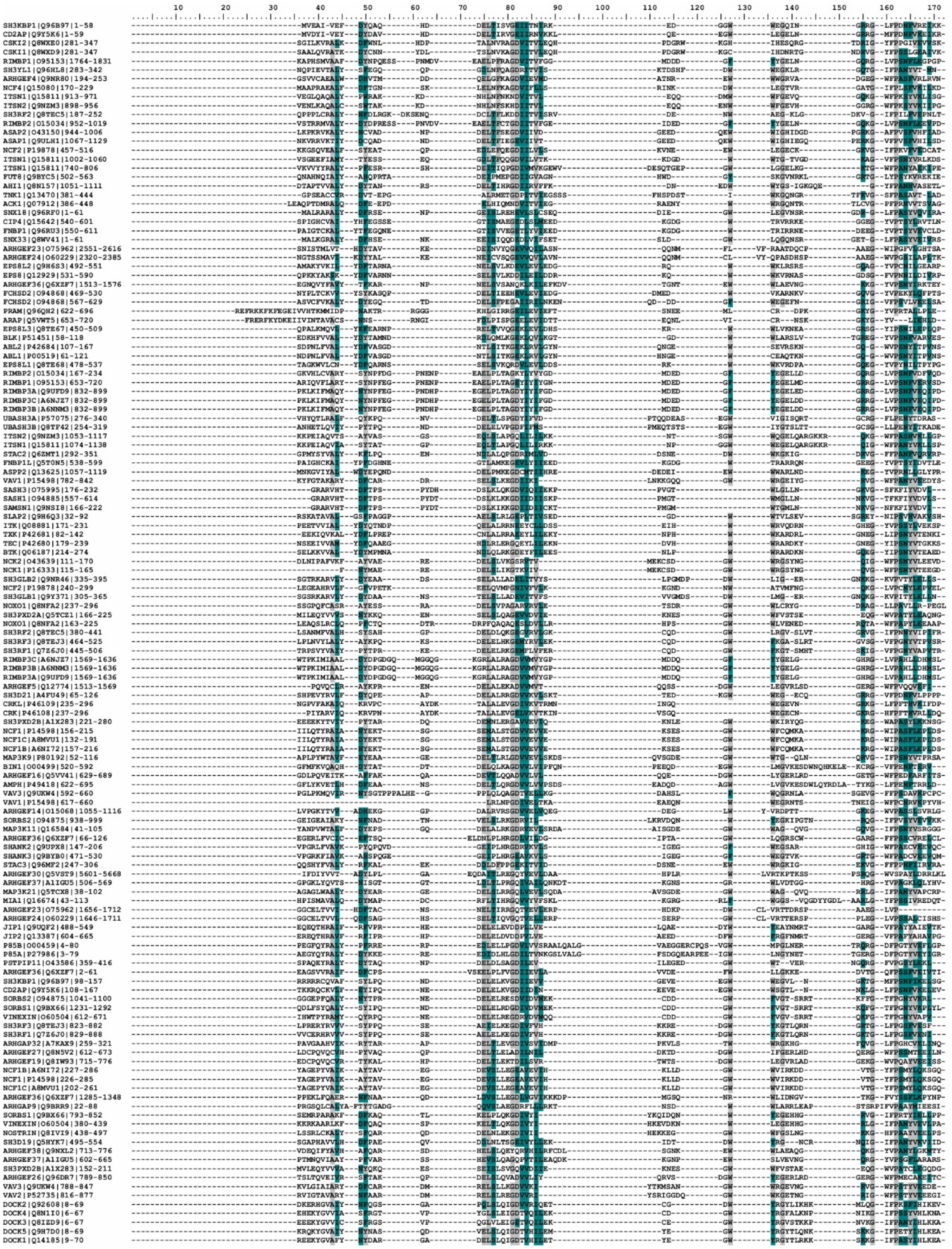

Figure S2. Alignment of SH3 domain sequences. The multiple sequence alignment of the SH3 domains was generated using the BioEdit program by CLUSTALW. Amino acids that are either identical or similar are indicated by gray and green shading, respectively. Gaps are shown as dashed lines.

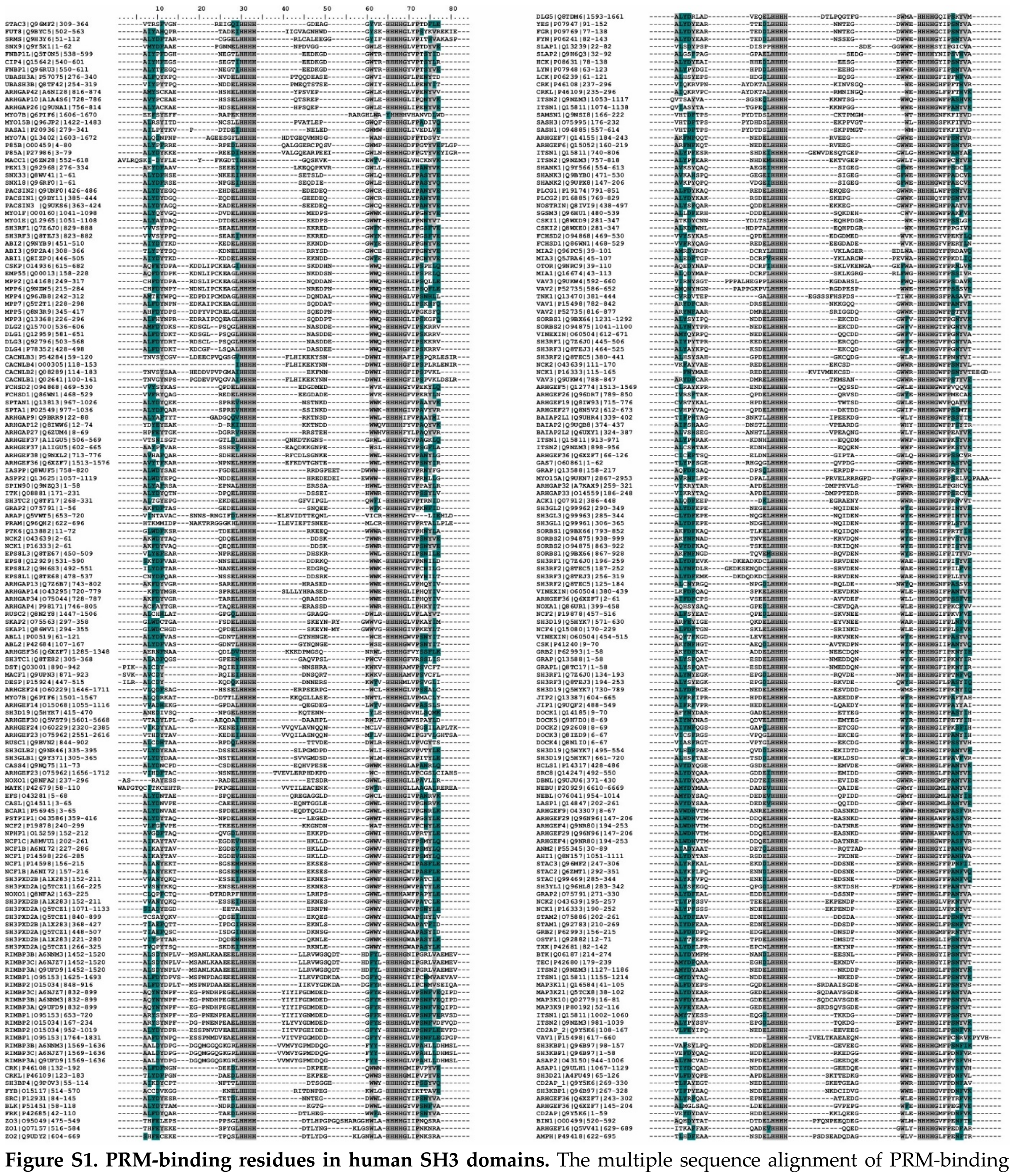

Figure S1. PRM-binding residues in human SH3 domains. The multiple sequence alignment of PRM-binding residues in SH3 domains is generated using ClustalW multiple alignments in BioEdit 7.2.5 software. Amino acids that are either identical or similar are shaded in gray and green, respectively. H-repeats indicate deleted parts of the SH3 domains.

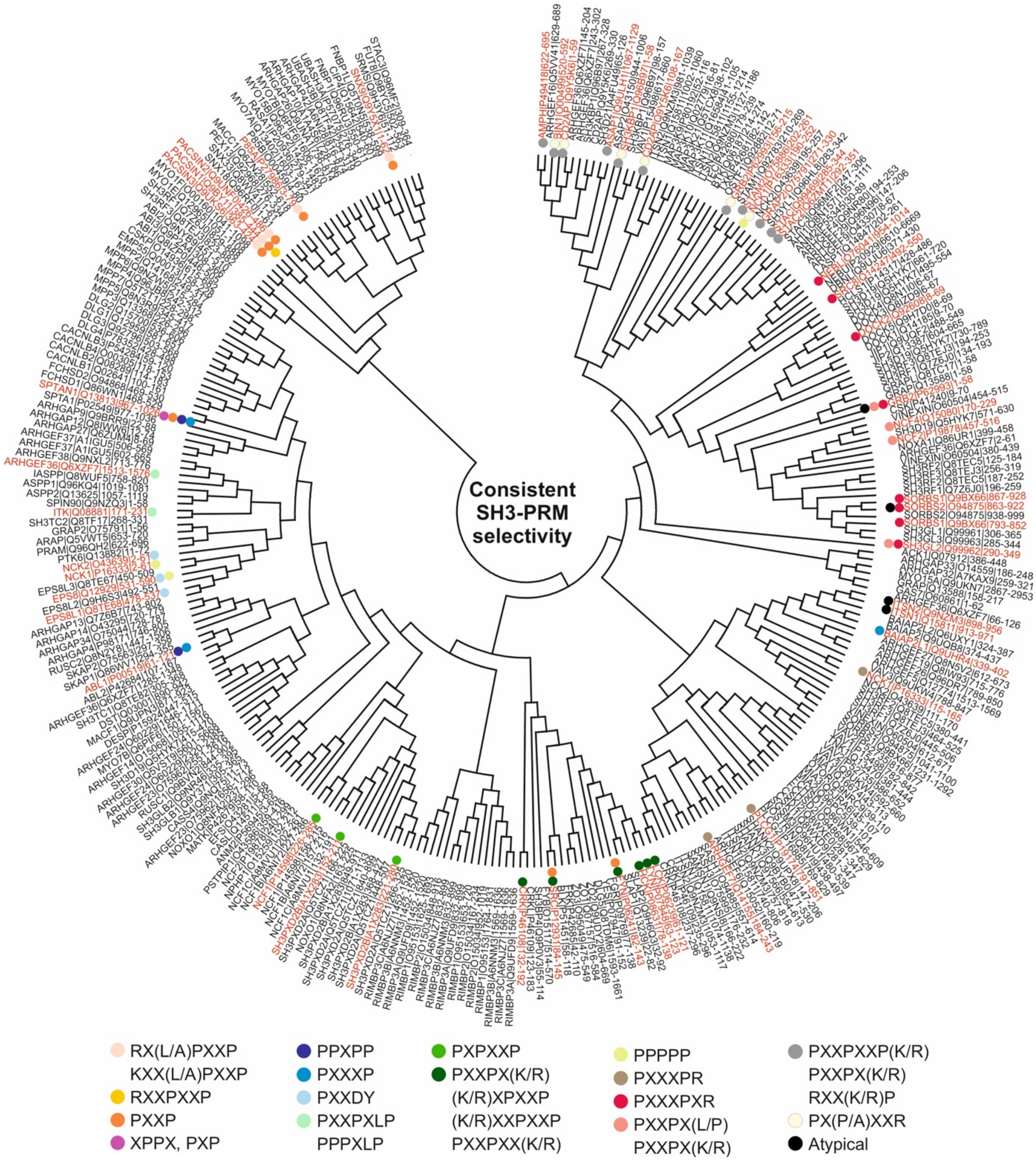

Figure S2. Exploring evolutionary relationships of PRM-interacting residues in SH3 domains. To construct the phylogenetic tree (tree #2), we meticulously examined PRM-interacting residues derived from 298 human SH3 domains, using the MEGA software (version 10.2.6). Using structural and biochemical data from SH3 domains (detailed in Tables S3 and S4), the graphical representation in the tree illustrates interactions between PRMs and their corresponding SH3s. Specific PRM preferences of SH3 domains are highlighted by colored circles, while the related SH3 domains are emphasized in red. Remarkably, the PRMs exhibit clustering patterns consistent with established SH3 domain families, allowing us to systematically categorize them into ten distinct families, each associated with specific PRMs, as shown below (Figure 1).

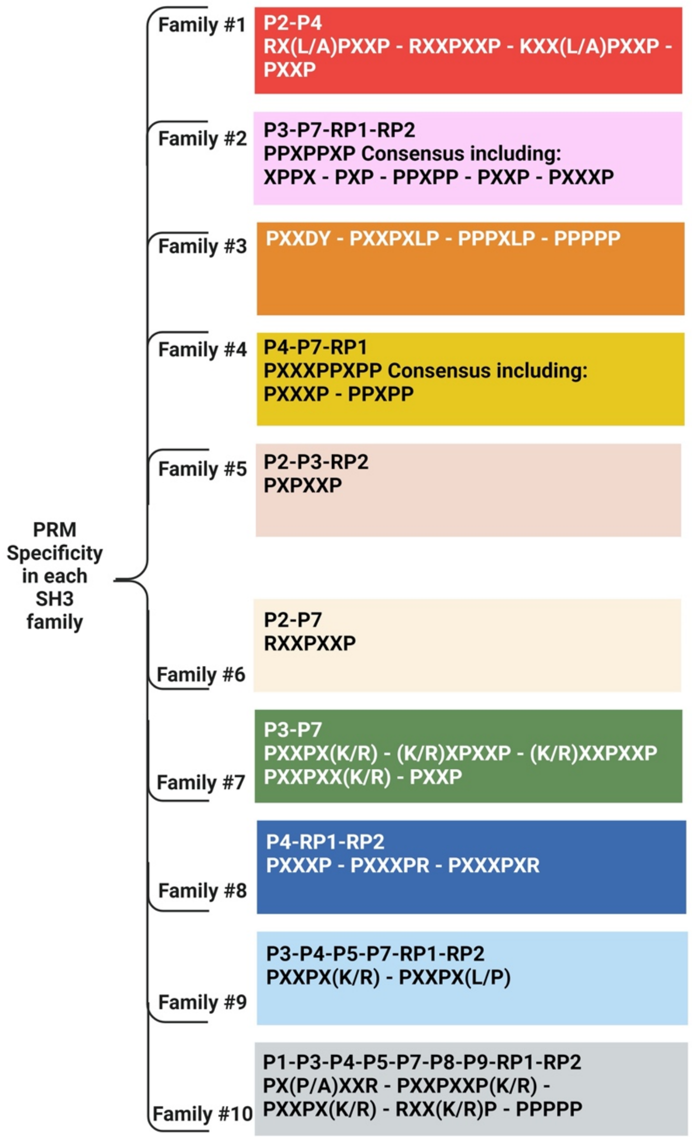

Figure S5. Analysis of SH3-PRM interaction specificity across different SH3 domain families within the human proteome. The top line illustrates the specificity of PRMs interacting with individual SH3 domain families represented by SH3 representatives from P1 to P10 and RP1 to RP2. The lower line delineates the specificity of the PRM motif within each family by evaluating structural and functional analyses of SH3 domains associated with PRMs as documented in published data (Tables S3 and S4).

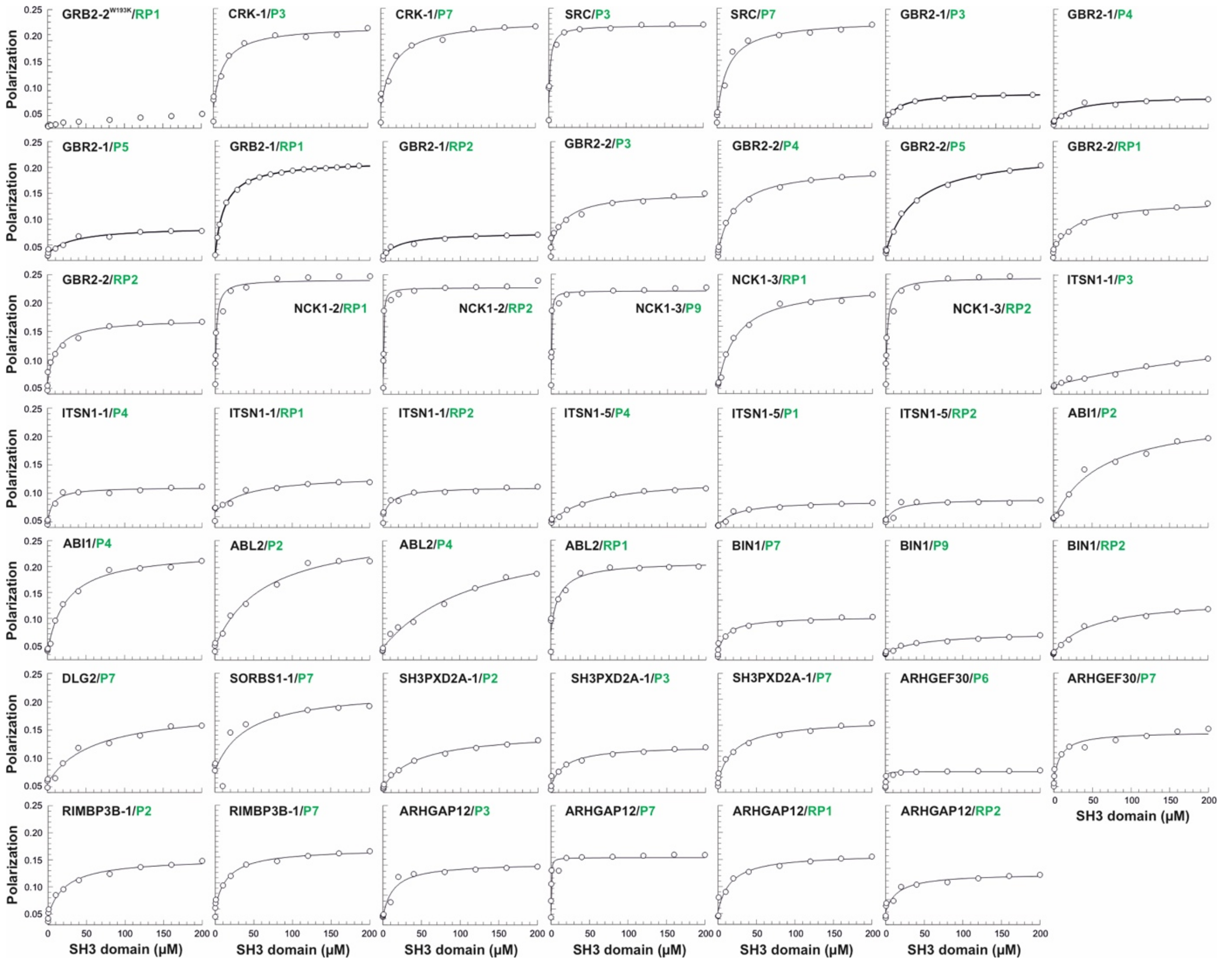

Figure S6. Interactions of the SH3 domains with fluorescent PRPs measured by fluorescence polarization. Fluorescent peptides (0.2 μM) were titrated with increasing concentrations of the corresponding SH3 domains. GRB2-2^{W193K}, defective in the binding of PRPs such as RP1, was used as a negative control as previously described [94]. The x-axis represents SH3 domain concentrations as GST fusion proteins in μM, while the y-axis represents fluorescence polarization. The equilibrium dissociation constants (K_d) for the respective measurements were determined by fitting the titration curves to a quadratic ligand-binding equation (solid lines). All K_d values are summarized in Figure 2B and Table S6. Error bars are derived from the fitting errors.

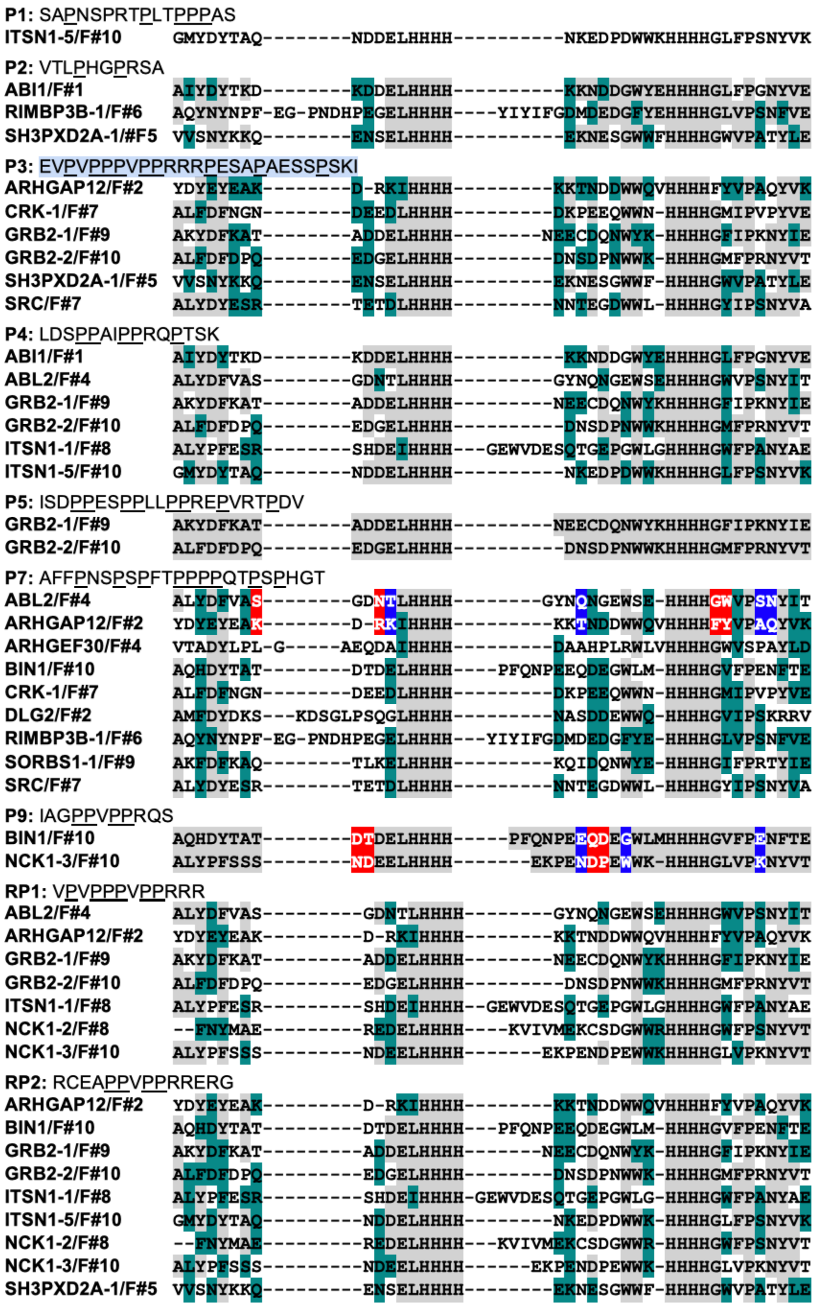

Figure S7. Sequence alignment of PRM-binding residues in representative SH3 domains interacting with specific PRPs. Conserved residues crucial for these interactions are highlighted. H-repeats indicate deleted portions of the SH3 domains. The proteins are also assigned to their respective families according to Figure 1. Residues in red (Set-1) and in blue (Set-2) are non-conserved residues and are the subjects of mutational analysis.

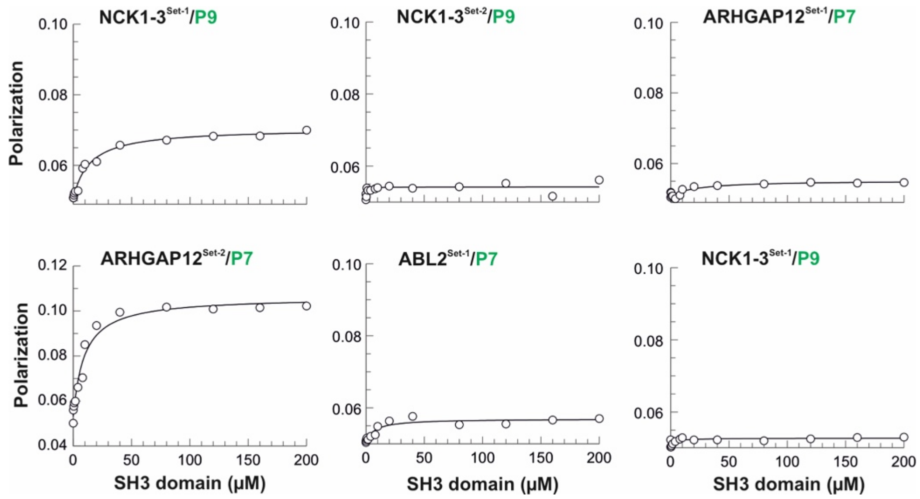

Figure S8. Mutational analysis of the SH3-fluorescent PRPs interactions using fluorescence polarization. Fluorescent peptides (0.2 µM) were titrated with increasing concentrations of the corresponding SH3 domain mutants (see Figure 3A and Table S1). The x-axis represents SH3 domain concentrations as GST fusion proteins in µM, while the y-axis represents fluorescence polarization. The equilibrium dissociation constants (K_d) for the respective measurements were determined by fitting the titration curves to a quadratic ligand-binding equation (solid lines). All K_d values are summarized in Figure 3B. Error bars are derived from the fitting errors.

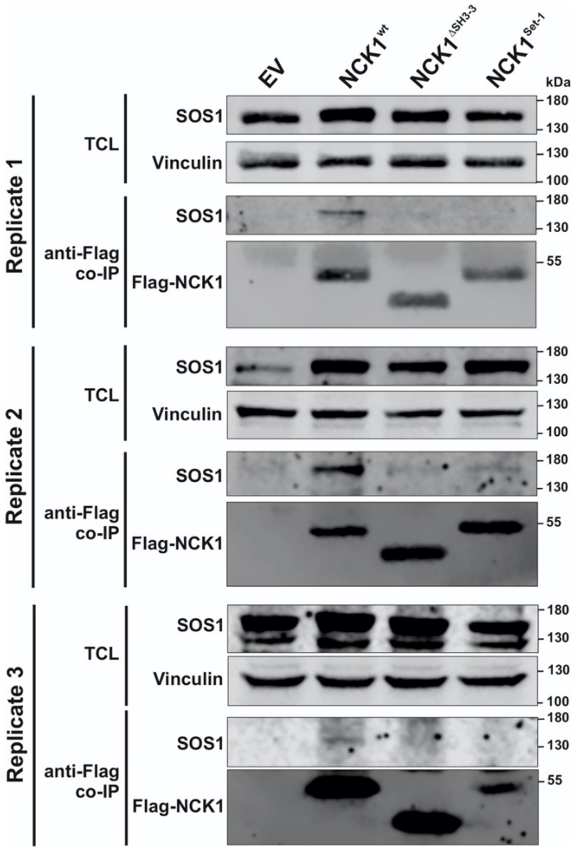

Figure S9. Co-immunoprecipitation of NCK1 with SOS1 in CHO-K1 cells. Experimental replicates of co-immunoprecipitation (co-IP) assays were conducted in CHO-K1 cells, co-transfected with HA-tagged SOS1 and Flag-tagged NCK1^{wt}, NCK1$^{\Delta SH3\text{-}3}$, and NCK1$^{Set\text{-}1}$. Co-IP was performed using anti-Flag beads to investigate potential interactions between NCK1 and SOS1 in the cellular context. The immunoblot analysis was performed using anti-SOS1 (1:1000; #sc-256, Santa Cruz) and anti-Flag (#F742, Sigma) antibodies. All three replicates showed co-immunoprecipitation of SOS1 with NCK1^{wt} but not NCK1$^{\Delta SH3\text{-}3}$, or NCK1$^{Set\text{-}1}$.

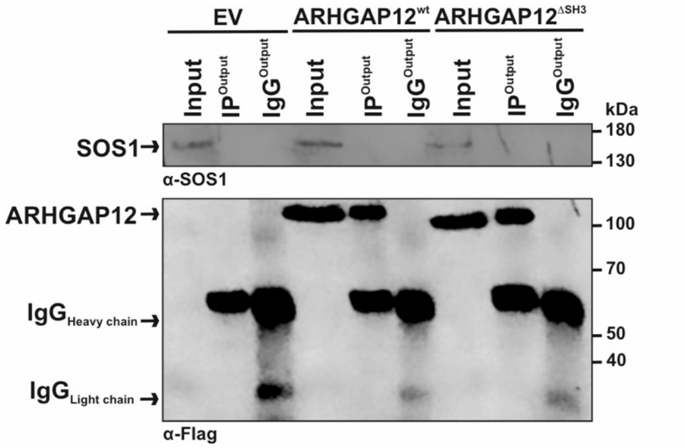

Figure S10. No co-immunoprecipitation of SOS1 with ARHGAP12 in CHO-K1 cells. Co-immunoprecipitation (co-IP) assay was conducted in CHO-K1 cells, co-transfected with HA-tagged SOS1 and Flag-tagged ARHGAP12^{wt}, and ARHGAP12$^{\Delta SH3-3}$. Co-IP was performed using protein A beads to investigate potential interactions between ARHGAP12 and SOS1 in the cellular context. Lysates from these transfected cells were subjected to Co-IP using anti-Flag (1:50; #F3165, Sigma) and anti-IgG (1:50; # sc-2025, Santa Cruz) antibodies coupled to protein A beads. The immunoblot analysis was performed using anti-SOS1 (1:1000; #sc-256, Santa Cruz) and anti-Flag (#F742, Sigma) antibodies. immunoblot analysis using anti-SOS1 (1:1000; #sc-256, Santa Cruz) and anti-Flag (#F742, Sigma) antibodies revealed no interaction neither ARHGAP12^{wt} nor ARHGAP12$^{\Delta SH3}$ with HA-SOS1.

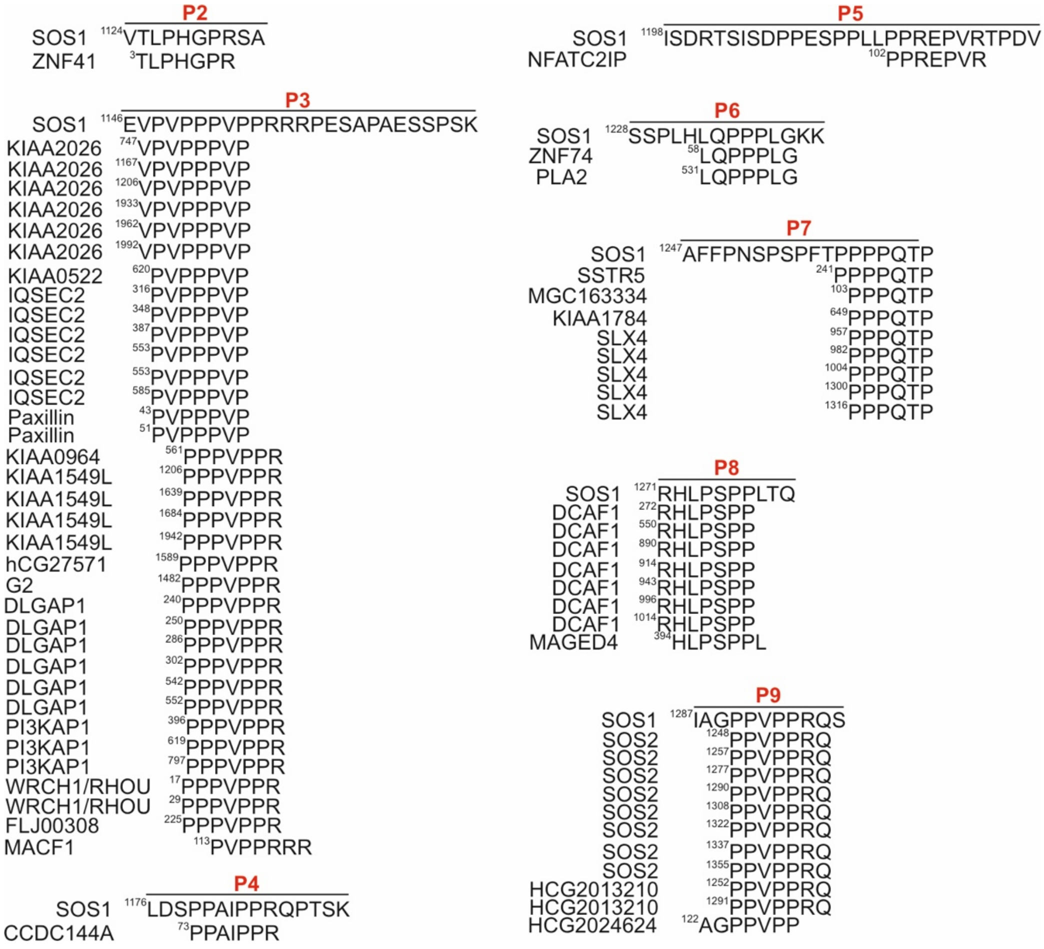

Figure S11. SOS1 homologous PRM sequences found in other human proteins. BLAST searches associated with each SOS1 PRD peptide identified homologous sequences in other human proteins (See also Table S7 and Figure 4).

References

1. Goretzki, B., N. A. Glogowski, E. Diehl, E. Duchardt-Ferner, C. Hacker, R. Gaudet, and U. A. Hellmich. "Structural Basis of Trpv4 n Terminus Interaction with Syndapin/Pacsin1-3 and Pip(2)." *Structure* 26, no. 12 (2018): 1583-93.e5.
2. Tossavainen, Helena, Hasan Uğurlu, Mikael Karjalainen, Maarit Hellman, Lina Antenucci, Riku Fagerlund, Kalle Saksela, and Perttu Permi. "Structure of Snx9 Sh3 in Complex with a Viral Ligand Reveals the Molecular Basis of Its Unique Specificity for Alanine-Containing Class I Sh3 Motifs." *Structure* 30, no. 6 (2022): 828-39. e6.
3. Batra-Safferling, R., J. Granzin, S. Mödder, S. Hoffmann, and D. Willbold. "Structural Studies of the Phosphatidylinositol 3-Kinase (Pi3k) Sh3 Domain in Complex with a Peptide Ligand: Role of the Anchor Residue in Ligand Binding." *Biol Chem* 391, no. 1 (2010): 33-42.
4. Casares, S., E. Ab, H. Eshuis, O. Lopez-Mayorga, N. A. van Nuland, and F. Conejero-Lara. "The High-Resolution Nmr Structure of the R21a Spc-Sh3:P41 Complex: Understanding the Determinants of Binding Affinity by Comparison with Abl-Sh3." *BMC Struct Biol* 7 (2007): 22.
5. Gushchina, L. V., A. G. Gabdulkhakov, S. V. Nikonov, and V. V. Filimonov. "High-Resolution Crystal Structure of Spectrin Sh3 Domain Fused with a Proline-Rich Peptide." *J Biomol Struct Dyn* 29, no. 3 (2011): 485-95.
6. Takeuchi, Koh, Hailin Yang, Elise Ng, Sungh-youk Park, Zhen-Yu J Sun, Ellis L Reinherz, and Gerhard Wagner. "Structural and Functional Evidence That Nck Interaction with Cd3ε Regulates T-Cell Receptor Activity." *Journal of molecular biology* 380, no. 4 (2008): 704-16.
7. Aitio, Olli, Maarit Hellman, Tapio Kesti, Iivari Kleino, Olga Samuilova, Kimmo Pääkkönen, Helena Tossavainen, Kalle Saksela, and Perttu Permi. "Structural Basis of Pxxdy Motif Recognition in Sh3 Binding." *Journal of Molecular Biology* 382, no. 1 (2008): 167-78.
8. Polle, Lilia, Luciano A Rigano, Rowan Julian, Keith Ireton, and Wolf-Dieter Schubert. "Structural Details of Human Tuba Recruitment by Inlc of Listeria Monocytogenes Elucidate Bacterial Cell-Cell Spreading." *Structure* 22, no. 2 (2014): 304-14.
9. Cámara-Artigas, A., A. Palencia, J. C. Martínez, I. Luque, J. A. Gavira, and J. M. García-Ruiz. "Crystallization by Capillary Counter-Diffusion and Structure Determination of the N114a Mutant of the Sh3 Domain of Abl Tyrosine Kinase Complexed with a High-Affinity Peptide Ligand." *Acta Crystallogr D Biol Crystallogr* 63, no. Pt 5 (2007): 646-52.
10. Grover, Prerna, Haibin Shi, Matthew Baumgartner, Carlos J Camacho, and Thomas E Smithgall. "Fluorescence Polarization Screening Assays for Small Molecule Allosteric Modulators of Abl Kinase Function." *PLoS One* 10, no. 7 (2015): e0133590.
11. Palencia, A., A. Camara-Artigas, M. T. Pisabarro, J. C. Martinez, and I. Luque. "Role of Interfacial Water Molecules in Proline-Rich Ligand Recognition by the Src Homology 3 Domain of Abl." *J Biol Chem* 285, no. 4 (2010): 2823-33.
12. Musacchio, Andrea, Matti Saraste, and Matthias Wilmanns. "High-Resolution Crystal Structures of Tyrosine Kinase Sh3 Domains Complexed with Proline-Rich Peptides." *Nature structural biology* 1, no. 8 (1994): 546-51.
13. Groemping, Y., K. Lapouge, S. J. Smerdon, and K. Rittinger. "Molecular Basis of Phosphorylation-Induced Activation of the Nadph Oxidase." *Cell* 113, no. 3 (2003): 343-55.
14. Ogura, K., I. Nobuhisa, S. Yuzawa, R. Takeya, S. Torikai, K. Saikawa, H. Sumimoto, and F. Inagaki. "Nmr Solution Structure of the Tandem Src Homology 3 Domains of P47phox Complexed with a P22phox-Derived Proline-Rich Peptide." *J Biol Chem* 281, no. 6 (2006): 3660-8.
15. Wu, Xiaodong, Beatrice Knudsen, Stephan M. Feller, Jie Zheng, Andrej Sali, David Cowburn, Hidesaburo Hanafusa, and John Kuriyan. "Structural Basis for the Specific Interaction of Lysine-Containing Proline-Rich Peptides with the N-Terminal Sh3 Domain of C-Crk." *Structure* 3, no. 2 (1995): 215-26.
16. Bhatt, Veer S, Danyun Zeng, Inna Krieger, James C Sacchettini, and Jae-Hyun Cho. "Binding Mechanism of the N-Terminal Sh3 Domain of Crkii and Proline-Rich Motifs in Cabl." *Biophysical journal* 110, no. 12 (2016): 2630-41.
17. Nguyen, Jack T., Christoph W. Turck, Fred E. Cohen, Ronald N. Zuckermann, and Wendell A. Lim. "Exploiting the Basis of Proline Recognition by Sh3 and Ww Domains: Design of N-Substituted Inhibitors." *Science* 282, no. 5396 (1998): 2088-92.
18. Schmidt, Holger, Silke Hoffmann, Tuyen Tran, Matthias Stoldt, Thomas Stangler, Katja Wiesehan, and Dieter Willbold. "Solution Structure of a Hck Sh3 Domain Ligand Complex Reveals Novel Interaction Modes." *Journal of molecular biology* 365, no. 5 (2007): 1517-32.
19. Camara-Artigas, Ana, Emilia Ortiz-Salmeron, Montserrat Andujar-Sánchez, Julio Bacarizo, and Jose Manuel Martin-Garcia. "The Role of Water Molecules in the Binding of Class I and Ii Peptides to the Sh3 Domain of

the Fyn Tyrosine Kinase." *Acta Crystallographica Section F: Structural Biology Communications* 72, no. 9 (2016): 707-12.

20. Renzoni, D. A., D. J. Pugh, G. Siligardi, P. Das, C. J. Morton, C. Rossi, M. D. Waterfield, I. D. Campbell, and J. E. Ladbury. "Structural and Thermodynamic Characterization of the Interaction of the Sh3 Domain from Fyn with the Proline-Rich Binding Site on the P85 Subunit of Pi3-Kinase." *Biochemistry* 35, no. 49 (1996): 15646-53.
21. Martin-Garcia, J. M., I. Luque, J. Ruiz-Sanz, and A. Camara-Artigas. "The Promiscuous Binding of the Fyn Sh3 Domain to a Peptide from the Ns5a Protein." *Acta Crystallogr D Biol Crystallogr* 68, no. Pt 8 (2012): 1030-40.
22. Schweimer, K., S. Hoffmann, F. Bauer, U. Friedrich, C. Kardinal, S. M. Feller, B. Biesinger, and H. Sticht. "Structural Investigation of the Binding of a Herpesviral Protein to the Sh3 Domain of Tyrosine Kinase Lck." *Biochemistry* 41, no. 16 (2002): 5120-30.
23. Feng, S., C. Kasahara, R. J. Rickles, and S. L. Schreiber. "Specific Interactions Outside the Proline-Rich Core of Two Classes of Src Homology 3 Ligands." *Proc Natl Acad Sci U S A* 92, no. 26 (1995): 12408-15.
24. Bacarizo, J., and A. Camara-Artigas. "Atomic Resolution Structures of the C-Src Sh3 Domain in Complex with Two High-Affinity Peptides from Classes I and Ii." *Acta Crystallogr D Biol Crystallogr* 69, no. Pt 5 (2013): 756-66.
25. Ghose, R., A. Shekhtman, M. J. Goger, H. Ji, and D. Cowburn. "A Novel, Specific Interaction Involving the Csk Sh3 Domain and Its Natural Ligand." *Nat Struct Biol* 8, no. 11 (2001): 998-1004.
26. Feng, Sibo, Tarun M. Kapoor, Fumiyuki Shirai, Andrew P. Combs, and Stuart L. Schreiber. "Molecular Basis for the Binding of Sh3 Ligands with Non-Peptide Elements Identified by Combinatorial Synthesis." *Chemistry & Biology* 3, no. 8 (1996): 661-70.
27. Feng, S., J. K. Chen, H. Yu, J. A. Simon, and S. L. Schreiber. "Two Binding Orientations for Peptides to the Src Sh3 Domain: Development of a General Model for Sh3-Ligand Interactions." *Science* 266, no. 5188 (1994): 1241-7.
28. Bacarizo, J., S. Martínez-Rodríguez, and A. Cámara-Artigas. "Structure of the C-Src-Sh3 Domain in Complex with a Proline-Rich Motif of Ns5a Protein from the Hepatitis C Virus." *J Struct Biol* 189, no. 1 (2015): 67-72.
29. Teyra, Joan, Haiming Huang, Shobhit Jain, Xinyu Guan, Aiping Dong, Yanli Liu, Wolfram Tempel, Jinrong Min, Yufeng Tong, and Philip M Kim. "Comprehensive Analysis of the Human Sh3 Domain Family Reveals a Wide Variety of Non-Canonical Specificities." *Structure* 25, no. 10 (2017): 1598-610. e3.
30. Mott, Helen R, Daniel Nietlispach, Katrina A Evetts, and Darerca Owen. "Structural Analysis of the Sh3 Domain of B-Pix and Its Interaction with A-P21 Activated Kinase (Pak)." *Biochemistry* 44, no. 33 (2005): 10977-83.
31. Jozic, Daniela, Nayra Cárdenes, Yonathan Lissanu Deribe, Gabriel Moncalián, Daniela Hoeller, Yvonne Groemping, Ivan Dikic, Katrin Rittinger, and Jerónimo Bravo. "Cbl Promotes Clustering of Endocytic Adaptor Proteins." *Nature structural & molecular biology* 12, no. 11 (2005): 972-79.
32. Janz, Jay M, Thomas P Sakmar, and K Christopher Min. "A Novel Interaction between Atrophin-Interacting Protein 4 and B-P21-Activated Kinase-Interactive Exchange Factor Is Mediated by an Sh3 Domain." *Journal of Biological Chemistry* 282, no. 39 (2007): 28893-903.
33. Desrochers, Guillaume, Laurent Cappadocia, Mathieu Lussier-Price, Anh-Tien Ton, Riham Ayoubi, Adrian Serohijos, James G Omichinski, and Annie Angers. "Molecular Basis of Interactions between Sh3 Domain-Containing Proteins and the Proline-Rich Region of the Ubiquitin Ligase Itch." *Journal of Biological Chemistry* 292, no. 15 (2017): 6325-38.
34. Hoelz, A., J. M. Janz, S. D. Lawrie, B. Corwin, A. Lee, and T. P. Sakmar. "Crystal Structure of the Sh3 Domain of Betapix in Complex with a High Affinity Peptide from Pak2." *J Mol Biol* 358, no. 2 (2006): 509-22.
35. Aitio, Olli, Maarit Hellman, Arunas Kazlauskas, Didier F Vingadassalom, John M Leong, Kalle Saksela, and Perttu Permi. "Recognition of Tandem Pxxp Motifs as a Unique Src Homology 3-Binding Mode Triggers Pathogen-Driven Actin Assembly." *Proceedings of the National Academy of Sciences* 107, no. 50 (2010): 21743-48.
36. Deng, L., C. A. Velikovsky, C. P. Swaminathan, S. Cho, and R. A. Mariuzza. "Structural Basis for Recognition of the T Cell Adaptor Protein Slp-76 by the Sh3 Domain of Phospholipase Cgamma1." *J Mol Biol* 352, no. 1 (2005): 1-10.
37. Vidal, Michel, Nathalie Goudreau, Fabrice Cornille, Didier Cussac, Edith Gincel, and Christiane Garbay. "Molecular and Cellular Analysis of Grb2 Sh3 Domain Mutants: Interaction with Sos and Dynamin." *Journal of molecular biology* 290, no. 3 (1999): 717-30.
38. Wittekind, M., C. Mapelli, V. Lee, V. Goldfarb, M. S. Friedrichs, C. A. Meyers, and L. Mueller. "Solution Structure of the Grb2 N-Terminal Sh3 Domain Complexed with a Ten-Residue Peptide Derived from Sos: Direct Refinement against Noes, J-Couplings and 1h and 13c Chemical Shifts." *J Mol Biol* 267, no. 4 (1997): 933-52.
39. Hanawa-Suetsugu, Kyoko, Mutsuko Kukimoto-Niino, Chiemi Mishima-Tsumagari, Ryogo Akasaka, Noboru Ohsawa, Shun-ichi Sekine, Takuhiro Ito, Naoya Tochio, Seizo Koshiba, and Takanori Kigawa. "Structural

Basis for Mutual Relief of the Rac Guanine Nucleotide Exchange Factor Dock2 and Its Partner Elmo1 from Their Autoinhibited Forms." *Proceedings of the National Academy of Sciences* 109, no. 9 (2012): 3305-10.
40. Kami, Keiichiro, Ryu Takeya, Hideki Sumimoto, and Daisuke Kohda. "Diverse Recognition of Non-Pxxp Peptide Ligands by the Sh3 Domains from P67phox, Grb2 and Pex13p." *The EMBO Journal* 21, no. 16 (2002): 4268-76.
41. Massenet, Claire, Sylvie Chenavas, Claudine Cohen-Addad, Marie-Claire Dagher, Gérard Brandolin, Eva Pebay-Peyroula, and Franck Fieschi. "Effects of P47phox C Terminus Phosphorylations on Binding Interactions with P40phox and P67phox: Structural and Functional Comparison of P40phox and P67phox Sh3 Domains." *Journal of Biological Chemistry* 280, no. 14 (2005): 13752-61.
42. Hashimoto, Shigeru, Mayumi Hirose, Ari Hashimoto, Masaki Morishige, Atsuko Yamada, Harumi Hosaka, Ken-ichi Akagi, Eiji Ogawa, Chitose Oneyama, and Tsutomu Agatsuma. "Targeting Amap1 and Cortactin Binding Bearing an Atypical Src Homology 3/Proline Interface for Prevention of Breast Cancer Invasion and Metastasis." *Proceedings of the National Academy of Sciences* 103, no. 18 (2006): 7036-41.
43. Liu, W., S. M. MacGrath, A. J. Koleske, and T. J. Boggon. "Lysozyme Contamination Facilitates Crystallization of a Heterotrimeric Cortactin-Arg-Lysozyme Complex." *Acta Crystallogr Sect F Struct Biol Cryst Commun* 68, no. Pt 2 (2012): 154-8.
44. Gehmlich, Katja, Nikos Pinotsis, Katrin Hayeß, Peter FM van der Ven, Hendrik Milting, Aly El Banayosy, Reiner Körfer, Matthias Wilmanns, Elisabeth Ehler, and Dieter O Fürst. "Paxillin and Ponsin Interact in Nascent Costameres of Muscle Cells." *Journal of molecular biology* 369, no. 3 (2007): 665-82.
45. Zhao, Debiao, Xuejuan Wang, Junhui Peng, Chongyuan Wang, Fudong Li, Qianqian Sun, Yibo Zhang, Jiahai Zhang, Gang Cai, and Xiaobing Zuo. "Structural Investigation of the Interaction between the Tandem Sh3 Domains of C-Cbl-Associated Protein and Vinculin." *Journal of structural biology* 187, no. 2 (2014): 194-205.
46. Eulitz, Stefan, Florian Sauer, Marie-Cecile Pelissier, Prisca Boisguerin, Sibylle Molt, Julia Schuld, Zacharias Orfanos, Rudolf A Kley, Rudolf Volkmer, and Matthias Wilmanns. "Identification of Xin-Repeat Proteins as Novel Ligands of the Sh3 Domains of Nebulin and Nebulette and Analysis of Their Interaction During Myofibril Formation and Remodeling." *Molecular biology of the cell* 24, no. 20 (2013): 3215-26.
47. Liao, Tsung-Jen, Hyunbum Jang, Ruth Nussinov, and David Fushman. "High-Affinity Interactions of the Nsh3/Csh3 Domains of Grb2 with the C-Terminal Proline-Rich Domain of Sos1." *Journal of the American Chemical Society* 142, no. 7 (2020): 3401-11.
48. Harkiolaki, M., T. Tsirka, M. Lewitzky, P. C. Simister, D. Joshi, L. E. Bird, E. Y. Jones, N. O'Reilly, and S. M. Feller. "Distinct Binding Modes of Two Epitopes in Gab2 That Interact with the Sh3c Domain of Grb2." *Structure* 17, no. 6 (2009): 809-22.
49. Rouka, Evgenia, Philip C Simister, Melanie Janning, Joerg Kumbrink, Tassos Konstantinou, João RC Muniz, Dhira Joshi, Nicola O'Reilly, Rudolf Volkmer, and Brigitte Ritter. "Differential Recognition Preferences of the Three Src Homology 3 (Sh3) Domains from the Adaptor Cd2-Associated Protein (Cd2ap) and Direct Association with Ras and Rab Interactor 3 (Rin3)." *Journal of Biological Chemistry* 290, no. 42 (2015): 25275-92.
50. Moncalián, Gabriel, Nayra Cárdenes, Yonathan Lissanu Deribe, Mercedes Spínola-Amilibia, Ivan Dikic, and Jerónimo Bravo. "Atypical Polyproline Recognition by the Cms N-Terminal Src Homology 3 Domain." *Journal of Biological Chemistry* 281, no. 50 (2006): 38845-53.
51. Hologne, Maggy, François-Xavier Cantrelle, Gwladys Riviere, Florence Guillière, Xavier Trivelli, and Olivier Walker. "Nmr Reveals the Interplay among the Amsh Sh3 Binding Motif, Stam2, and Lys63-Linked Diubiquitin." *Journal of molecular biology* 428, no. 22 (2016): 4544-58.
52. Kaneko, Tomonori, Takashi Kumasaka, Tadashi Ganbe, Takao Sato, Keiji Miyazawa, Naomi Kitamura, and Nobuo Tanaka. "Structural Insight into Modest Binding of a Non-Pxxp Ligand to the Signal Transducing Adaptor Molecule-2 Src Homology 3 Domain." *Journal of Biological Chemistry* 278, no. 48 (2003): 48162-68.
53. Pineda-Lucena, A., C. S. Ho, D. Y. Mao, Y. Sheng, R. C. Laister, R. Muhandiram, Y. Lu, B. T. Seet, S. Katz, T. Szyperski, L. Z. Penn, and C. H. Arrowsmith. "A Structure-Based Model of the C-Myc/Bin1 Protein Interaction Shows Alternative Splicing of Bin1 and C-Myc Phosphorylation Are Key Binding Determinants." *J Mol Biol* 351, no. 1 (2005): 182-94.
54. Lewitzky, M., M. Harkiolaki, M. C. Domart, E. Y. Jones, and S. M. Feller. "Mona/Gads Sh3c Binding to Hematopoietic Progenitor Kinase 1 (Hpk1) Combines an Atypical Sh3 Binding Motif, R/Kxxk, with a Classical Pxxp Motif Embedded in a Polyproline Type Ii (Ppii) Helix." *J Biol Chem* 279, no. 27 (2004): 28724-32.
55. Harkiolaki, M., M. Lewitzky, R. J. Gilbert, E. Y. Jones, R. P. Bourette, G. Mouchiroud, H. Sondermann, I. Moarefi, and S. M. Feller. "Structural Basis for Sh3 Domain-Mediated High-Affinity Binding between Mona/Gads and Slp-76." *Embo j* 22, no. 11 (2003): 2571-82.
56. Liu, Q., D. Berry, P. Nash, T. Pawson, C. J. McGlade, and S. S. Li. "Structural Basis for Specific Binding of the Gads Sh3 Domain to an Rxxk Motif-Containing Slp-76 Peptide: A Novel Mode of Peptide Recognition." *Mol Cell* 11, no. 2 (2003): 471-81.

57. Dimasi, N. "Crystal Structure of the C-Terminal Sh3 Domain of the Adaptor Protein Gads in Complex with Slp-76 Motif Peptide Reveals a Unique Sh3-Sh3 Interaction." *Int J Biochem Cell Biol* 39, no. 1 (2007): 109-23.
58. Jia, X., L. Lin, S. Xu, L. Li, Z. Wei, C. Yu, and F. Niu. "Crystal Structure of the Sh3 Domain of Asap1 in Complex with the Proline Rich Motif (Prm) of Mical1 Reveals a Unique Sh3/Prm Interaction Mode." *Int J Mol Sci* 24, no. 2 (2023).
59. Wong King Yuen, Siobhan M., Marta Campiglio, Ching-Chieh Tung, Bernhard E. Flucher, and Filip Van Petegem. "Structural Insights into Binding of Stac Proteins to Voltage-Gated Calcium Channels." *Proceedings of the National Academy of Sciences* 114, no. 45 (2017): E9520-E28.
60. Desrochers, Guillaume, Mathieu Lussier-Price, James G. Omichinski, and Annie Angers. "Multiple Src Homology 3 Binding to the Ubiquitin Ligase Itch Conserved Proline-Rich Region." *Biochemistry* 54, no. 50 (2015): 7345-54.
61. Goretzki, Benedikt, Nina A Glogowski, Erika Diehl, Elke Duchardt-Ferner, Carolin Hacker, Rachelle Gaudet, and Ute A Hellmich. "Structural Basis of Trpv4 N Terminus Interaction with Syndapin/Pacsin1-3 and Pip2." *Structure* 26, no. 12 (2018): 1583-93. e5.
62. Yu, Hongtao, James K Chen, Sibo Feng, David C Dalgarno, Andrew W Brauer, and Stuart L Schrelber. "Structural Basis for the Binding of Proline-Rich Peptides to Sh3 Domains." *Cell* 76, no. 5 (1994): 933-45.
63. Gushchina, LV, AG Gabdulkhakov, and VV Filimonov. "Design and Structural Thermodynamic Studies of the Chimeric Protein Derived from Spectrin Sh3 Domain." *Molecular biology* 43 (2009): 444-52.
64. Banjade, Sudeep, Qiong Wu, Anuradha Mittal, William B Peeples, Rohit V Pappu, and Michael K Rosen. "Conserved Interdomain Linker Promotes Phase Separation of the Multivalent Adaptor Protein Nck." *Proceedings of the National Academy of Sciences* 112, no. 47 (2015): E6426-E35.
65. Mongioví, A. M., P. R. Romano, S. Panni, M. Mendoza, W. T. Wong, A. Musacchio, G. Cesareni, and P. P. Di Fiore. "A Novel Peptide-Sh3 Interaction." *Embo j* 18, no. 19 (1999): 5300-9.
66. Andersen, Thorny Cesilie Bie, Per Eugen Kristiansen, Zsuzsa Huszenicza, Maria U. Johansson, Ramakrishna Prabhu Gopalakrishnan, Hanna Kjelstrup, Scott Boyken, Vibeke Sundvold-Gjerstad, Stine Granum, Morten Sørli, Paul Hoff Backe, D. Bruce Fulton, B. Göran Karlsson, Amy H. Andreotti, and Anne Spurkland. "The Sh3 Domains of the Protein Kinases Itk and Lck Compete for Adjacent Sites on T Cell–Specific Adapter Protein." *Journal of Biological Chemistry* 294, no. 42 (2019): 15480-94.
67. Pisabarro, MT, and L Serrano. "Rational Design of Specific High-Affinity Peptide Ligands for the Abl-Sh3 Domain." *Biochemistry* 35, no. 33 (1996): 10634-40.
68. Merő, B., K. Koprivanacz, A. Cserkaszky, L. Radnai, V. Vas, G. Kudlik, G. Gógl, P. Sok, L. Póti Á, B. Szeder, L. Nyitray, A. Reményi, M. Geiszt, and L. Buday. "Characterization of the Intramolecular Interactions and Regulatory Mechanisms of the Scaffold Protein Tks4." *Int J Mol Sci* 22, no. 15 (2021).
69. Knudsen, B. S., J. Zheng, S. M. Feller, J. P. Mayer, S. K. Burrell, D. Cowburn, and H. Hanafusa. "Affinity and Specificity Requirements for the First Src Homology 3 Domain of the Crk Proteins." *Embo j* 14, no. 10 (1995): 2191-8.
70. Sarkar, P., C. Reichman, T. Saleh, R. B. Birge, and C. G. Kalodimos. "Proline Cis-Trans Isomerization Controls Autoinhibition of a Signaling Protein." *Mol Cell* 25, no. 3 (2007): 413-26.
71. Matsuda, Michiyuki, Satoshi Ota, Ryuji Tanimura, Haruki Nakamura, Koozi Matuoka, Tadaomi Takenawa, Kazuo Nagashima, and Takeshi Kurata. "Interaction between the Amino-Terminal Sh3 Domain of Crk and Its Natural Target Proteins*." *Journal of Biological Chemistry* 271, no. 24 (1996): 14468-72.
72. Lee, C. H., B. Leung, M. A. Lemmon, J. Zheng, D. Cowburn, J. Kuriyan, and K. Saksela. "A Single Amino Acid in the Sh3 Domain of Hck Determines Its High Affinity and Specificity in Binding to Hiv-1 Nef Protein." *Embo j* 14, no. 20 (1995): 5006-15.
73. Morton, Craig J, David JR Pugh, Emma LJ Brown, Jan D Kahmann, Debora AC Renzoni, and Iain D Campbell. "Solution Structure and Peptide Binding of the Sh3 Domain from Human Fyn." *Structure* 4, no. 6 (1996): 705-14.
74. Bauer, Finn, and Heinrich Sticht. "A Proline to Glycine Mutation in the Lck Sh3-Domain Affects Conformational Sampling and Increases Ligand Binding Affinity." *FEBS letters* 581, no. 8 (2007): 1555-60.
75. Zeng, Longhui, Ivan Palaia, Anđela Šarić, and Xiaolei Su. "Plcγ1 Promotes Phase Separation of T Cell Signaling Components." *Journal of Cell Biology* 220, no. 6 (2021).
76. Aitio, O., M. Hellman, A. Kazlauskas, D. F. Vingadassalom, J. M. Leong, K. Saksela, and P. Permi. "Recognition of Tandem Pxxp Motifs as a Unique Src Homology 3-Binding Mode Triggers Pathogen-Driven Actin Assembly." *Proc Natl Acad Sci U S A* 107, no. 50 (2010): 21743-8.
77. Goudreau, N, F Cornille, M Duchesne, F Parker, B Tocque, C Garbay, and BP Roques. "Nmr Structure of the N-Terminal Sh3 Domain of Grb2 and Its Complex with a Proline-Rich Peptide from Sos." *Nature structural biology* 1, no. 12 (1994): 898-907.

78. McDonald, Caleb B, Kenneth L Seldeen, Brian J Deegan, and Amjad Farooq. "Structural Basis of the Differential Binding of the Sh3 Domains of Grb2 Adaptor to the Guanine Nucleotide Exchange Factor Sos1." *Archives of biochemistry and biophysics* 479, no. 1 (2008): 52-62.
79. ———. "Sh3 Domains of Grb2 Adaptor Bind to Pxψpxr Motifs within the Sos1 Nucleotide Exchange Factor in a Discriminate Manner." *Biochemistry* 48, no. 19 (2009): 4074-85.
80. Grabs, Detlev, Vladimir I Slepnev, Zhou Songyang, Carol David, Mary Lynch, Lewis C Cantley, and Pietro De Camilli. "The Sh3 Domain of Amphiphysin Binds the Proline-Rich Domain of Dynamin at a Single Site That Defines a New Sh3 Binding Consensus Sequence." *Journal of Biological Chemistry* 272, no. 20 (1997): 13419-25.
81. Lasorsa, Alessia, Krishnendu Bera, Idir Malki, Elian Dupré, François-Xavier Cantrelle, Hamida Merzougui, Davy Sinnaeve, Xavier Hanoulle, Jozef Hritz, and Isabelle Landrieu. "Conformation and Affinity Modulations by Multiple Phosphorylation Occurring in the Bin1 Sh3 Domain Binding Site of the Tau Protein Proline-Rich Region." *Biochemistry* 62, no. 11 (2023): 1631-42.
82. Hutchings, Nicholas J, Nicholas Clarkson, Robert Chalkley, A Neil Barclay, and Marion H Brown. "Linking the T Cell Surface Protein Cd2 to the Actin-Capping Protein Capz Via Cms and Cin85." *Journal of Biological Chemistry* 278, no. 25 (2003): 22396-403.
83. Fang, Nancy N, Gerard T Chan, Mang Zhu, Sophie A Comyn, Avinash Persaud, Raymond J Deshaies, Daniela Rotin, Joerg Gsponer, and Thibault Mayor. "Rsp5/Nedd4 Is the Main Ubiquitin Ligase That Targets Cytosolic Misfolded Proteins Following Heat Stress." *Nature cell biology* 16, no. 12 (2014): 1227-37.
84. Peil, Lauri, Agata L Starosta, Jürgen Lassak, Gemma C Atkinson, Kai Virumäe, Michaela Spitzer, Tanel Tenson, Kirsten Jung, Jaanus Remme, and Daniel N Wilson. "Distinct Xppx Sequence Motifs Induce Ribosome Stalling, Which Is Rescued by the Translation Elongation Factor Ef-P." *Proceedings of the National Academy of Sciences* 110, no. 38 (2013): 15265-70.
85. Hupp, Ted R, and Malcolm Walkinshaw. "Multienzyme Assembly of a P53 Transcription Complex." *Nature structural & molecular biology* 14, no. 10 (2007): 885-87.
86. Dornan, David, Harumi Shimizu, Lindsay Burch, Amanda J Smith, and Ted R Hupp. "The Proline Repeat Domain of P53 Binds Directly to the Transcriptional Coactivator P300 and Allosterically Controls DNA-Dependent Acetylation of P53." *Molecular and cellular biology* 23, no. 23 (2003): 8846-61.
87. Guruprasad, L, V Dhanaraj, D Timm, TL Blundell, I Gout, and MD Waterfield. "The Crystal Structure of the N-Terminal Sh3 Domain of Grb2." *Journal of molecular biology* 248, no. 4 (1995): 856-66.
88. Tan, Soon-Heng, Willy Hugo, Wing-Kin Sung, and See-Kiong Ng. "A Correlated Motif Approach for Finding Short Linear Motifs from Protein Interaction Networks." *BMC bioinformatics* 7, no. 1 (2006): 502.
89. Rader, Katherine, Robert A Orlando, Xiaojing Lou, and Marilyn Gist Farquhar. "Characterization of Ankra, a Novel Ankyrin Repeat Protein That Interacts with the Cytoplasmic Domain of Megalin." *Journal of the American Society of Nephrology* 11, no. 12 (2000): 2167-78.
90. Menon, Rajesh P, and R Colin Hughes. "Determinants in the N-Terminal Domains of Galectin-3 for Secretion by a Novel Pathway Circumventing the Endoplasmic Reticulum–Golgi Complex." *European journal of biochemistry* 264, no. 2 (1999): 569-76.
91. Beltrao, Pedro, and Luis Serrano. "Comparative Genomics and Disorder Prediction Identify Biologically Relevant Sh3 Protein Interactions." *PLoS computational biology* 1, no. 3 (2005).
92. Kowanetz, Katarzyna, Koraljka Husnjak, Daniela Holler, Marcin Kowanetz, Philippe Soubeyran, Dianne Hirsch, Mirko HH Schmidt, Kresimir Pavelic, Pietro De Camilli, and Paul A Randazzo. "Cin85 Associates with Multiple Effectors Controlling Intracellular Trafficking of Epidermal Growth Factor Receptors." *Molecular biology of the cell* 15, no. 7 (2004): 3155-66.
93. D'erchia, AM, G Pesole, A Tullo, C Saccone, and E Sbisa. "Guinea Pig P53 Mrna: Identification of New Elements in Coding and Untranslated Regions and Their Functional and Evolutionary Implications." *Genomics* 58, no. 1 (1999): 50-64.
94. Kazemein Jasemi, Neda S., Christian Herrmann, Eva Magdalena Estirado, Lothar Gremer, Dieter Willbold, Luc Brunsveld, Radovan Dvorsky, and Mohammad R. Ahmadian. "The Intramolecular Allostery of Grb2 Governing Its Interaction with Sos1 Is Modulated by Phosphotyrosine Ligands." *Biochemical Journal* 478, no. 14 (2021): 2793-809.
